# The Plant Phenology Ontology: A New Informatics Resource for Large-Scale Integration of Plant Phenology Data

**DOI:** 10.3389/fpls.2018.00517

**Published:** 2018-05-01

**Authors:** Brian J. Stucky, Rob Guralnick, John Deck, Ellen G. Denny, Kjell Bolmgren, Ramona Walls

**Affiliations:** ^1^Florida Museum of Natural History, University of Florida, Gainesville, FL, United States; ^2^Berkeley Natural History Museums, University of California, Berkeley, Berkeley, CA, United States; ^3^USA National Phenology Network, The University of Arizona, Tucson, AZ, United States; ^4^Unit for Field-based Forest Research, Swedish University of Agricultural Sciences, Lammhult, Sweden; ^5^CyVerse, The University of Arizona, Tucson, AZ, United States

**Keywords:** plant phenology, USA National Phenology Network, Pan-European Phenology Network, ontology, data integration, knowledge representation, semantic data

## Abstract

Plant phenology – the timing of plant life-cycle events, such as flowering or leafing out – plays a fundamental role in the functioning of terrestrial ecosystems, including human agricultural systems. Because plant phenology is often linked with climatic variables, there is widespread interest in developing a deeper understanding of global plant phenology patterns and trends. Although phenology data from around the world are currently available, truly global analyses of plant phenology have so far been difficult because the organizations producing large-scale phenology data are using non-standardized terminologies and metrics during data collection and data processing. To address this problem, we have developed the Plant Phenology Ontology (PPO). The PPO provides the standardized vocabulary and semantic framework that is needed for large-scale integration of heterogeneous plant phenology data. Here, we describe the PPO, and we also report preliminary results of using the PPO and a new data processing pipeline to build a large dataset of phenology information from North America and Europe.

## Introduction

Plant phenology – the timing of plant life-cycle events, such as leaf bud burst, flowering, and fruiting – has cascading effects on multiple levels of biological organization, from individuals to ecosystems. Phenology not only affects the fitness of individual plants, it also affects the fitness of organisms that depend on them, which, in terrestrial ecosystems, includes virtually all animals. Thus, changes in plant phenology can negatively impact demography, cause rapid evolutionary shifts, and result in agricultural losses (Reilly et al., 1996; Chmielewski et al., 2004; Visser and Both, 2005; Franks et al., 2007; Willis et al., 2008; Miller-Rushing et al., 2010; Anderson et al., 2012; McKinney et al., 2012; Miller-Struttmann et al., 2015). Furthermore, the phenological responses of plants are known to be highly responsive to environmental drivers and thus strongly influenced by climate change (Parmesan and Yohe, 2003; Menzel et al., 2006; Cleland et al., 2007; IPCC, 2008; Wolkovich et al., 2012; Chuine and Régnière, 2017). Advancing our understanding of the drivers of phenological response can provide insight into future states of species distributions (Durant et al., 2005; Chuine, 2010), biogeochemistry (Richardson et al., 2010), and ecosystem services such as pollination (Memmott et al., 2007; McKinney et al., 2012; Høye et al., 2013; Kharouba et al., 2018). Therefore, increasing scientific understanding of relationships between phenology and the structure and function of ecosystems can help inform adaptive management of natural resources (Walther, 2010; Bellard et al., 2012; Enquist et al., 2014; Pacifici et al., 2015).

Despite the importance of understanding phenology for managing biodiversity and ecosystem services, studying plant phenology at transcontinental or global scales remains very challenging (Cook et al., 2012; Wolkovich et al., 2012; Davies et al., 2013; Kharouba et al., 2018). This is not merely due to a lack of data; in fact, multiple national- and continent-scale phenology data sources are already available, such as the USA National Phenology Network (USA-NPN)^1^ (Rosemartin et al., 2014), the U.S.-based National Ecological Observatory Network (NEON)^2^ (Elmendorf et al., 2016), the Pan-European Phenology Database (PEP725)^3^ (Koch et al., 2010; Templ et al., 2018), Canada PlantWatch^4^ (Beaubien and Hamann, 2011), SeasonWatch in India^5^, ClimateWatch in Australia^6^, and numerous others. Further, there is also a rich, yet scattered, legacy phenology record available from herbarium specimens (Davis et al., 2015). The problem is that curators of plant phenology data often use project-specific terminologies and metrics during data collection and data processing, because a comprehensive, standardized, formal description of plant phenology and phenological data has so far not been available. The end outcome is considerable inefficiency as data and knowledge producers build similar but non-interoperable end products.

If these data interoperability issues could be overcome, it would be possible to assemble data resources for studying plant phenology at the broadest geographic, temporal, and phylogenetic extents (e.g., Cook et al., 2012; Davies et al., 2013). The mere existence of such a global phenology data resource would provide almost immediate answers to key questions regarding phenology patterns, such as: How similar are phenological patterns in relation to latitude across continents? How have global phenology patterns changed over time? Can we assemble a full understanding of the phenology patterns of widespread species across their ranges? With climate data and other complementary datasets increasingly available at global scale, more complex, process-driven questions would also be in reach.

Here, we introduce the Plant Phenology Ontology (PPO), a formal semantic framework for plant phenology data that can help address the shortcomings of current practice and pave the way for integration of field- and herbarium-based phenology data on a global scale. The PPO provides a vocabulary of clearly defined terms for describing plant phenology and phenological data, and it also provides a rigorous description logic-based foundation for these terms so that phenology data can be used directly with knowledge representation systems that support automated reasoning. We also report preliminary results of ongoing efforts to use the PPO to combine disparate phenology datasets from USA-NPN, NEON, and PEP725, and we show that these integrated data can be used in transcontinental phenology analyses.

## Methods

### Design Goals

Prior to beginning PPO development, we identified six guiding design goals. Specifically, the PPO needed to be: (1) Broad enough to cover vegetative and reproductive development stages of plants; (2) Applicable to (nearly) all gymnosperms and angiosperms; (3) Suitable for both single plants and populations of plants; (4) Compatible with the data and data collection methods of USA-NPN and NEON, which use status-based monitoring, and PEP725, which uses event-based monitoring (Denny et al., 2014); (5) Compatible with protocols for capturing phenology data from herbarium specimens (Yost et al., 2018); and (5) Interoperable with existing OBO Foundry library (Smith et al., 2007) ontologies, especially the Plant Ontology (PO) (Cooper et al., 2013) and the Biological Collections Ontology (BCO) (Walls et al., 2014a). These design goals were intended to ensure broad applicability and reusability of the PPO and data based on the PPO, and they guided the methods used for the actual development work, which we describe next.

### Phenology Data and Ontology Scope

Most phenology data come from one of four sources: ground-based observations, satellite or airborne remote sensing, automated digital repeat photography, and historical plant specimens in museum collections. Other sources of information about plant phenology, such as data from experimental treatments and flux tower measurements, will not be considered here for the sake of brevity. Ground-based observations typically pertain to either individual plants or local populations of plants, and they are collected from professional and citizen scientists, researchers’ field notes, and agricultural and forestry records. In contrast, satellite and airborne remote sensing approaches capture relatively coarse, aggregated measures of leaf reflectance which are used to determine “greenness”. While these images can provide information about phenology patterns at the canopy level across large geographic areas, flowering time is largely ignored, understory plants can confound greenness measurements, and it is challenging to fully reconcile greenness with ground-based observations (Schwartz and Reed, 1999). Digital repeat photography (Sonnentag et al., 2012), of which the PhenoCam project is perhaps the best-known example (Richardson et al., 2009, 2018; Brown et al., 2016), bridges some of the data gaps between ground-based observations and satellite imagery. Digital repeat photography uses “phenocams”, which are stationary digital cameras that automatically take photographs of a single community of plants at regular time intervals. The photographs are subsequently analyzed to make inferences about the timing of “greening up” or flowering. Finally, herbaria are an important source of historical phenology information, and phenological observation of herbarium specimens is roughly analogous to ground-based observation of living plants (Primack et al., 2004; Bolmgren and Lönnberg, 2005; Calinger et al., 2013). Although millions of digital records from U.S. herbaria are now available through repositories such as iDigBio, these records rarely include explicit information about phenology. Thus, historical phenology data from herbarium collections remains a largely untapped resource, although this is rapidly changing (Willis et al., 2017).

Ground-based observations and herbarium data are similar in that they derive from direct observation of individual plants or local groups of plants, and as such, they provide spatially fine-grained, direct information about plant phenology. Furthermore, most existing phenology data networks are geared towards these kinds of data. For these reasons, PPO development was focused on direct observation data with the goal of describing the phenology of single plants and populations of plants.

### Ontology Development

Prior to beginning formal ontology development, a preliminary discussion and data analysis workshop was held in January of 2016 at the USGS Powell Center in Fort Collins, CO, United States. This workshop included participants representing the organizations that collect and host many of the major sources of phenology data as well as scientists who use plant phenology data. A second workshop, focused on extracting phenology information from herbarium specimens, was held in March of 2016 at the University of California, Berkeley, and also helped inform subsequent ontology development (Yost et al., 2018).

Initial domain analysis and ontology design began by analyzing existing phenology data (primarily from USA-NPN and PEP725) and herbarium specimens to elucidate the classes (concepts) and relations (properties) needed to represent plant phenology and phenological data. The provisional classes and relations identified in these first analyses were then implemented and iteratively refined by attempting to map the data model of the PPO to the information contained in real phenology data from USA-NPN, NEON, and PEP725.

To map phenology data to the PPO, we examined all phenological terminology used by the USA-NPN, NEON, and PEP725 databases and evaluated whether each term and associated observations could be faithfully modeled using entities from the PPO. This exercise frequently revealed shortcomings of the PPO, some of which were relatively trivial (e.g., a missing class) and some of which required substantial design changes. Once the required changes were implemented in the PPO, we again attempted to map real phenology data to the PPO, and we repeated this evaluation/implementation cycle until the PPO could handle nearly all records contained in the USA-NPN, NEON, and PEP725 databases. The major exceptions were records in PEP725 that explicitly dealt with agricultural information, such as when a crop was harvested. Such information is outside the scope of the PPO, so we made no attempt to map these records or support them in the PPO.

The PPO was developed to conform to the principles of the OBO Foundry in order to promote integration with other ontologies for biodiversity data, especially the PO and BCO. To this end, classes and properties from other OBO Foundry ontologies, including the PO and BCO, were used whenever possible by importing them into the PPO. The Basic Formal Ontology (BFO) (Arp et al., 2015) was used as the upper-level ontology for the PPO.

The ontology development software OntoPilot^7^ (Stucky et al., in preparation) was used to implement new PPO classes and properties, to extract imported entities from other OBO ontologies, and to generate release versions of the PPO, including “reasoned” versions of the ontology with inferencing provided by the HermiT reasoner (Glimm et al., 2014). The PPO uses Manchester Syntax (Horridge and Patel-Schneider, 2012) for all description logic axioms and Web Ontology Language (OWL) (Hitzler et al., 2012) for all released versions of the ontology.

To help validate the PPO’s logical structure, we developed a test knowledge base containing instances of various “presence” classes with associated instances of ‘*measurement datum*’ (see section “Results”, below, for a description of these entities). The test knowledge base included both zero and non-zero count and percentage data values. During ontology development, we tested the PPO’s logical structure by using a reasoner and OntoPilot to materialize assertions that could be inferred from the test knowledge base, then comparing these inferred assertions to a set of expected assertions that had been generated by hand.

### Pipeline for Phenology Data Integration

To build a proof-of-concept knowledge base of PPO-integrated phenology data, we developed a highly customizable data processing pipeline that accepts raw phenology data from a provider, such as PEP725, and uses the PPO to convert it to a form suitable for inclusion in a common knowledge base. We will describe this pipeline in detail in a separate article; here, we merely provide a high-level overview. Source code and documentation for the pipeline are available at: https://github.com/biocodellc/ppo-data-pipeline.

The data processing pipeline comprised four distinct steps. First, the pipeline accepted source data in a variety of formats and converted all incoming data to a standard CSV (comma-separated values) text file format. Second, mappings from the source data to the PPO (see, “Ontology implementation”, above) were used to convert the data to a PPO-based, graph representation. Third, OntoPilot and a modified ELK reasoner (Kazakov et al., 2014) were used to add inferred facts about the phenology observations to the data graph. Fourth, and finally, the data graph was converted to a tabular format and loaded into an ElasticSearch database^8^ for data querying and retrieval (see proof-of-concept data portal^9^.

We used two methods to test that the data integration pipeline was correctly mapping and serving source phenology data. First, a series of automated tests was used to confirm that data were correctly mapped and processed in each stage of the pipeline. Second, we used USA-NPN’s Phenology Observation Portal^10^ to help verify the results of running the integration pipeline on raw USA-NPN data. We ran equivalent queries of the Phenology Observation Portal and the database generated by the integration pipeline, then manually compared matched pairs of result sets to confirm that they were the same.

### Sample Data Analyses

To analyze integrated USA-NPN, NEON, and PEP725 data, we examined leafing out dates for the genera *Acer* (maples) and *Quercus* (oaks) and flowering dates for the genera *Acer* and *Syringa* (lilacs). These genera were chosen because they occur in both Europe and North America, they each had a large overall number of records in the combined data set, and they each were well represented in data from both continents. To estimate leafing out dates, we used all observations of plants with the PPO trait ‘*true leaves present*’ that did not also have the trait ‘*senescing true leaves present*’, and to estimate flowering dates, we used all observations of plants with the PPO trait ‘*flowers present*’ that did not also have the trait ‘*senesced flowers present*’. All geographic locations (i.e., latitude and longitude) were rounded to a 0.1-degree grid, and the data were filtered to only keep the earliest relevant observation for each unique combination of grid cell and year. The filtered data were then analyzed and visualized using R (R Core Team, 2017). For most analyses, decades with fewer than 1,000 records were discarded. The only exception was the analysis of *Acer* leaf out times; in this case, because of a smaller total number of records, decades with fewer than 400 records were discarded. For visualizations that included distribution estimates, probability density functions were estimated using Gaussian kernel density estimation with the rule-of-thumb bandwidth based on the interquartile range as proposed by Silverman (1998).

## Results

The Plant Phenology Ontology, available at: http://purl.obolibrary.org/obo/ppo.owl, defines a total of 253 new classes, four new data properties, and seven new object properties. Source files and development versions of the PPO, including testing code, are available at the PPO’s git repository, https://github.com/PlantPhenoOntology/ppo. The PPO should be suitable for describing phenological data for nearly all gymnosperms and angiosperms, including crop plants, although agriculture-specific observations (e.g., planting and harvest) are not currently included in the PPO’s data model. The components of the PPO can be conceptually divided into three parts: (1) entities and axioms for modeling plant structures; (2) entities and axioms for modeling what we call “phenological traits”; and (3) entities and axioms for modeling observations of and data about phenological traits. To make the PPO data model as clear as possible, we will describe the general modeling approach for each of these parts in some detail. Ontology entity labels are written in italic typeface and enclosed in single quotes. To aid understanding the PPO data model, **Table 1** provides brief descriptions of some of the external ontology entities referenced by the PPO and used in the following descriptions.

**Table 1:** Key terms (i.e., ontology entities) used by the PPO that are imported from external ontologies.

Entity	Source	Definition and comments
Measurement datum	IAO	Defined as “an information content entity that is a recording of the output of a measurement such as produced by a device”. The IAO provides typical examples: “the recoding [sic] of the weight of a mouse”, “the recording of an observation of the behavior of the mouse”, and “the recording of the expression level of a gene as measured through the process of microarray experiment”.
Observing process	BCO	Defined as “a process in which a person or machine sees or detects a material entity and selects it as worthy of observation, and which has as output an information content entity about the selected material entity”.
Quality	PATO	Defined (rather opaquely) as “a dependent entity that inheres in a bearer by virtue of how the bearer is related to other entities”. Practically, this means that a ‘*quality*’ is something we typically view as a trait or characteristic of something else, such as the color of an apple, the shape of a wing, or, in the PPO, the presence of phenologically relevant structures on a plant.

*Only terms that are important for understanding the PPO description in the main text, and for which the meaning might not be readily apparent, are included. Key to source ontology abbreviations: BCO, Biological Collections Ontology (http://purl.obolibrary.org/obo/bco.owl), IAO, Information Artifact Ontology (http://purl.obolibrary.org/obo/iao.owl), PATO, Phenotype and Trait Ontology (http://purl.obolibrary.org/obo/pato.owl)*.

### Plant Structures

Information about in-situ plant phenology ultimately derives from information about physical structures on the plants themselves, such as leaves and flowers. Thus, the PPO includes a rich set of classes and axioms that describe phenologically relevant physical plant structures. All plant structure classes in the PPO ultimately descend from the PO class ‘*plant structure*’, which is defined as “An anatomical structure that is or was part of a plant, or was derived from a part of a plant”. The PO itself includes a large number of plant structure classes, and these are reused in the PPO whenever possible. Thus, the PPO only defines new plant structures that are currently not part of the PO. In many such cases, new plant structures defined in the PPO are phenologically informative special cases of plant structures in the PO and thus are not appropriate for inclusion in the PO, such as the PPO class ‘*senesced flower*’, which is a subclass of the PO class ‘*flower*’.

The PPO includes plant structure classes for shoot systems, leaves (including leaf buds), and reproductive structures for both gymnosperms and angiosperms (e.g., cones, flowers, and fruits). The PPO also includes logical axioms that define how the various plant structures are related to each other. These include subclass relationships (e.g., ‘*expanding true leaf*’ is a subclass of ‘*true leaf*’) and parthood relationships (e.g., a ‘*breaking leaf bud*’ ‘*has visible part*’ some instance of ‘*unfolding true leaf*’).

### Phenological Traits

Literature sources often discuss plant phenology in terms of “phenological stages” or “phenophases”, which are intended to denote phenologically important segments of a plant’s life cycle (Meier, 1997). Although it can be useful to think of plant phenology in terms of phenological stages, when we gather data about the phenology of a plant or group of plants, we do not really observe phenological stages, which usually occur over a period of days to month. Rather, we observe physical traits of plants that provide information about a plant’s phenology, such as whether a plant has leaves or ripe fruits. Because the goal of the PPO is to model information (i.e., data) about plant phenology, the PPO models phenology data in terms of physical traits of plants. This keeps the PPO’s data model close to how phenology data are actually obtained, and we also believe it will make the PPO more broadly useful to phenology researchers. There are no universally agreed-upon “phenological stages”, so modeling phenology data at the level of plant traits will make it easier for scientists to reuse phenology data for whatever purposes they require. The situation is a bit like that of a medical doctor examining a patient. A physician can observe signs or symptoms (i.e., traits) of a patient, such as body temperature or blood pressure, and these data might then be used to diagnose an illness or prescribe medication. Ontology of clinical data would probably not, however, include any attempts to automatically diagnose illnesses or prescribe medications. Similarly, the PPO provides a rich toolset for modeling data about plant phenology, but makes no attempt to “diagnose” phenological stages or any other high-level, conceptual views of plant phenology. Therefore, the PPO does not include any classes to define phenological stages or phases, but it does include a large number of classes and axioms that define “phenological traits” – the directly observable qualities of a plant that provide information about the plant’s phenology.

The top-level (i.e., most generic) PPO phenological trait class, and the parent class of all other phenological trait classes, is ‘*plant phenological trait*’, which is a subclass of ‘*quality*’ from the Phenotype and Trait Ontology (PATO)^11^ (see **Table 1**). A plant phenological trait is defined as “A ‘*quality*’ of a ‘*whole plant*’ that provides phenologically relevant information about the plant”. Phenological traits in the PPO are defined in terms of one or more plant structures associated with the trait. For instance, the trait ‘*true leaf presence*’ is defined as “A ‘*plant phenological trait*’ that is measured by the number of true leaves on a ‘*whole plant*”’. The PPO provides similar “presence” traits for many other plant structures, including shoot systems, leaves, and reproductive structures. The PPO also includes traits for abscised or removed (by an herbivore, e.g.) plant structures, such as ‘*abscised leaf presence*’, which is defined as “A ‘*plant phenological trait*’ that is measured by the number of true leaves that a ‘*whole plant*’ has abscised”.

**FIGURE 1:**
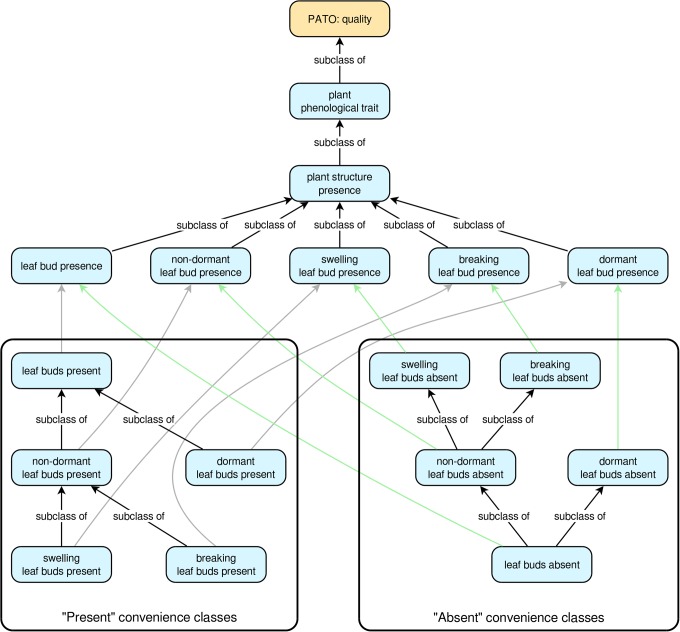
The trait class hierarchies for all Plant Phenology Ontology (PPO) trait classes pertaining to leaf buds. All arrows indicate subclass relationships, with gray arrows indicating subclass relationships between “present” classes and higher-level “presence” classes, and green arrows indicating subclass relationships between “absent” classes and higher-level “presence” classes. The “subclass of” labels for the gray and green arrows have been omitted to simplify the diagram. Note that subclass relationships for the “present” convenience classes are inverted for the “absent” convenience classes.

For each “presence” trait class, there is also a pair of convenience subclasses that describe the common qualitative cases of a given plant structure being either present or absent. So, for the trait ‘*true leaf presence*’, the PPO includes the convenience subclasses ‘*true leaves present*’ and ‘*true leaves absent*’. These present/absent convenience classes are arranged into hierarchies that facilitate automatic inferencing from specific to more general cases or vice versa. For the “present” classes, the most specific classes are at the tips of the hierarchy, whereas the hierarchy is reversed for the “absent” classes (**Figure 1**). An example should help clarify this. Suppose we know that some plant has one or more dormant leaf buds. This implies that the plant also has leaf buds in the more general sense. However, if we know the plant does not have dormant leaf buds, we cannot conclude that it lacks leaf buds entirely (e.g., it might have non-dormant leaf buds). On the other hand, if we know the plant lacks leaf buds in the general sense, then we can conclude it must also lack dormant leaf buds. Thus, in the PPO, ‘*dormant leaf buds present*’ is a subclass of ‘*leaf buds present*’ (if a plant has dormant leaf buds it must also have leaf buds in the general sense), but ‘*leaf buds absent*’ is a subclass of ‘*dormant leaf buds absent*’ (if a plant does not have any leaf buds, then it cannot have dormant leaf buds) (**Figure 1**). The class hierarchies for all present/absent convenience classes are inferred directly from the relationships of the plant structures on which they depend, thus ensuring that as long as the relationships among the plant structures are correct, inferences based on the present/absent trait classes will also be correct.

### Observations of Phenological Traits and Phenological Data

Phenological traits describe observable, phenologically relevant characteristics of plants, but they do not describe phenological data or how it is generated. The PPO therefore includes additional entities for modeling phenological data. Conceptually, the PPO views phenological data as the outcome of a process of observing one or more phenological traits. This is implemented with a class called ‘*phenology observing process*’, which is a subclass of ‘*observing processes*’ from the BCO (see **Table 1**). Roughly speaking, an ‘*observing process*’ involves observing some object (the process’s input) and producing some information artifact as a result of the observation (the process’s output). In the case of a ‘*phenology observing process*’, the input is some ‘*whole plant*’ (i.e., the plant that is observed), and the output is a ‘*measurement datum*’ [from the Information Artifact Ontology (IAO)^12^; see **Table 1**]. In the context of the PPO, measurement data can be either counts or percentages, and the PPO defines data properties to connect numerical values to instances of ‘*measurement datum*’. These data properties allow for uncertainty by specifying ranges of possible values. To connect data to the traits they measure, each instance of ‘*measurement datum*’ is asserted to be a ‘*quality measurement of*’ an instance of some ‘*plant phenological trait*’.

**FIGURE 2:**
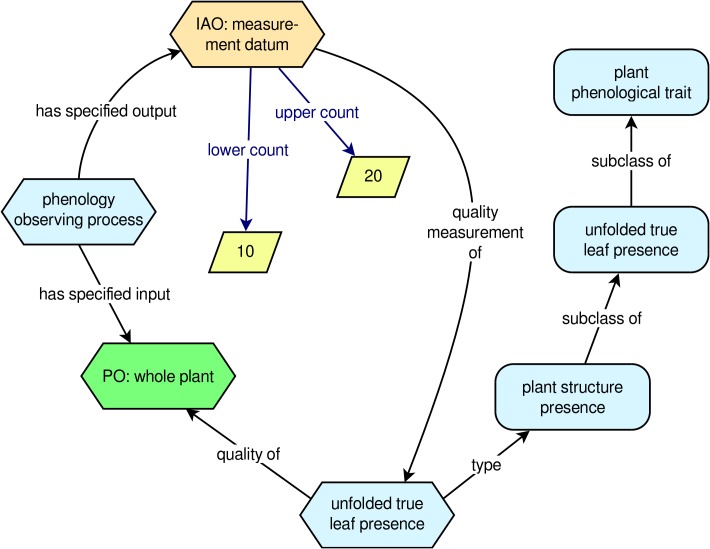
Example phenology observation modeled with the PPO. Rounded rectangles indicate ontology classes, hexagons indicate instances of classes, and rhomboids indicate literal numerical values.

To help clarify the PPO’s approach to modeling phenology observations and phenology data, it is useful to look at an example (**Figure 2**). Suppose a researcher observes a single sunflower plant and determines that there are between 10 and 20 unfolded true (i.e., non-cotyledon) leaves on the plant. From the PPO’s point of view, we have an instance of ‘*phenology observing process*’ with a ‘*whole plant*’ as its input (the sunflower that was observed) and an instance of ‘*measurement datum*’ as its output. The instance of ‘*measurement datum*’ is a ‘*quality measurement of*’ an instance of the trait ‘*unfolded true leaf presence*’, and the ‘*measurement datum*’ has a ‘*lower count*’ of 10 and an ‘*upper count*’ of 20. This approach, which corresponds with the way real phenology data are gathered by human observers, captures the following essential information about the datum: What plant was observed, the specific trait that was measured, and the outcome (i.e., the datum) of the observation. Additional information about the datum, such as the observation date and time, can easily be added using data properties from other ontologies, such as the BCO.

Logical axioms in the PPO allow for inferencing over the numerical values associated with instances of ‘*measurement datum*’ such that implicit instances of the present/absent convenience classes can be automatically recognized. Returning to the example of the number of unfolded true leaves on a sunflower, if we have an instance of ‘*measurement datum*’ that is a ‘*quality measurement of*’ an instance of the trait ‘*unfolded true leaf presence*’, and the ‘*measurement datum*’ has a ‘*lower count*’ of 10 and an ‘*upper count*’ of 20, logical axioms in the PPO would allow us to infer that this instance of ‘*unfolded true leaf presence*’ must also be an instance of the types ‘*unfolded true leaves present*’ and ‘*true leaves present*’. If, on the other hand, both ‘*lower count*’ and ‘*upper count*’ were zero, then the instance of ‘*unfolded true leaf presence*’ would be inferred to also have type ‘*unfolded true leaves absent*’. To put this another way, the PPO allows computers to understand that if a plant has one or more unfolded true leaves, the plant must also have the trait ‘*unfolded true leaves present*’ (and vice versa), and if a plant has zero unfolded true leaves, it must have the trait ‘*unfolded true leaves absent*’ (and vice versa). Thus, as this simple example shows, end users of the PPO can choose to explicitly model phenological data using a relatively small set of instances and assertions; much of the rest can be automatically inferred using a suitable reasoning engine. To put this another way, end users of the PPO can continue collecting phenological data however they like, and they can use any subset of the PPO that they feel best fits their data collection protocols.

### Phenology of Plant Populations and Communities

We have so far focused on modeling the phenology of individual plants, but the PPO could also be used to describe the phenology of plant populations and communities. In these cases, instances of plant phenological stages and traits would still pertain to individual plants, but the phenological stages and traits of the individual plants in a population or community could be used to describe the phenology of the population or community as a whole. This could be done either qualitatively or quantitatively. As an example of the former, we might state that at least some plants in a population have the quality ‘*leaves present*’. Or, to make a quantitative assessment, we might state that 80% of the plants in the population have the quality ‘*leaves present*’. Plant populations and communities can be represented by instances of subclasses of ‘*collection of organisms*’ from the Population and Community Ontology^13^ (Walls et al., 2014b), such as ‘*population*’ or ‘*ecological community*’.

### Preliminary Data Aggregation and Example Analyses

**FIGURE 3:**
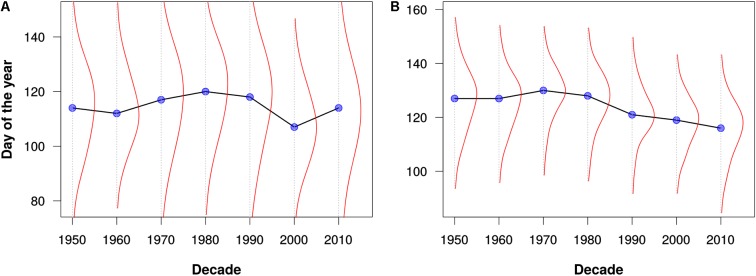
Leafing out times for **(A)** maples (*Acer*) and **(B)** oaks (*Quercus*) as estimated from all data for North America and Europe. The blue dots represent the median leafing out times for each decade. The red lines depict kernel density estimates of the distribution of leafing out times for each decade. Note that these graphs are intended merely to demonstrate the utility of the PPO for large-scale data integration and analysis; they should not be construed as robust phenological analyses.

**FIGURE 4:**
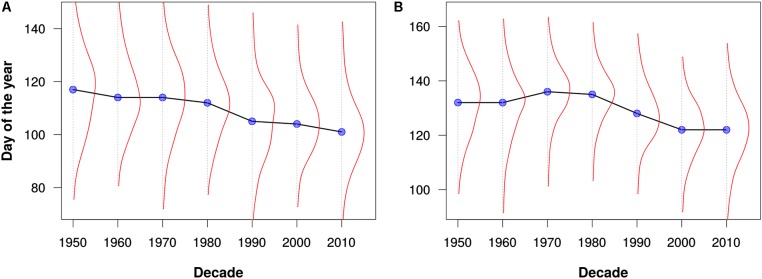
Flowering times for **(A)** maples (*Acer*) and **(B)** lilacs (*Syringa*) as estimated from all data for North America and Europe. The blue dots represent the median flowering times for each decade. The red lines depict kernel density estimates of the distribution of flowering out times for each decade. Note that these graphs are intended merely to demonstrate the utility of the PPO for large-scale data integration and analysis; they should not be construed as robust phenological analyses.

**FIGURE 5:**
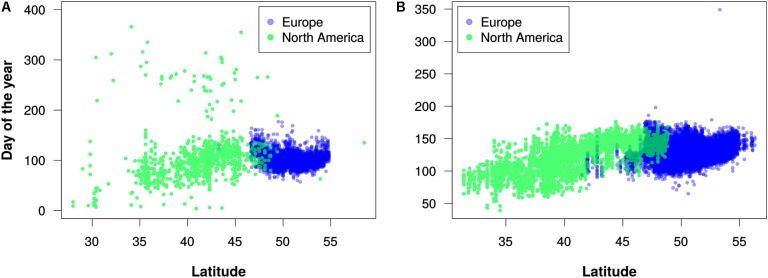
Latitudinal trends in flowering times for **(A)** maples (*Acer*) in the 2010s and **(B)** lilacs (*Syringa*) in the 1960s. Each dot represents the earliest estimated flowering time for a 0.1°-by-0.1° geographic grid cell. These decades were chosen because they had more even distribution of records between North America and Europe in comparison to other decades with available data. Note that these graphs are intended merely to demonstrate the utility of the PPO for large-scale data integration and analysis; they should not be construed as robust phenological analyses.

To test the PPO’s utility for large-scale integration of disparate phenological data, we used the PPO and data processing pipeline to combine all mappable records from three phenology monitoring networks with qualitatively different monitoring protocols: the status-based observations from the North American USA-NPN and NEON, and the event-based observations from the European PEP725. This produced an integrated knowledge base with a total of ∼19.9 million phenology observations, with 50.4% from PEP725, 43.8% from USA-NPN, and 5.8% from NEON. We then used these combined data for several simple, exploratory analyses of phenological patterns across Europe and North America. Specifically, we examined changes in leafing out and flowering times by decade and by geographic latitude. Example results for the genera *Acer* (maples), *Syringa* (lilicas), and *Quercus* (oaks) are shown in **Figures 3**–**5**. We wish to emphasize that these example analyses are not intended to be robust phenological studies; rather, they aim to demonstrate practical application of the PPO to real-world phenological data.

## Discussion

The PPO is the most comprehensive ontology available for modeling plant phenology data and the processes that generate them. It covers a broad range of phenological information, from the earliest visible growth of a plant, to reproductive activities, to the senescence of plant structures at the end of a growing season, and it is applicable to nearly all gymnosperms and angiosperms. Previously, phenology data sources have mostly been developed independently with little attention to interoperability, making unified analyses difficult at best or impracticable at worst. Our example analyses demonstrate that the PPO can be used to integrate these varied data sources and facilitate straightforward, cross-dataset queries and analyses. Although our examples were limited to USA-NPN, NEON, and PEP725 data, the PPO is sufficiently generic that it should be suitable for most new or extant phenology data generated from field-based observation of individual plants or plant communities, as well as at least some phenology data derived from herbarium specimens.

A major advantage of the PPO is its suite of logical axioms that allow for sophisticated, automated reasoning about phenology observations. In a knowledge base setting, these axioms provide a powerful mechanism for dataset integration and analysis. However, we wish to emphasize that end users of the PPO need not make use of these relatively advanced features. At its simplest, the PPO can be used as a basic controlled vocabulary that provides a set of clearly defined terms to describe plant phenology and phenology data. Data sets that use the PPO in this way will be poised for immediate integration with any other PPO-aware data sources, merely by using PPO terms as a common language for data exchange. This would be especially advantageous for new data providers because it could instantly expand the reach and reusability of their data. To facilitate using the PPO as a vocabulary for plant phenology data, we have developed a reference document that provides formal definitions, explanations, and examples for the most important PPO terms, available as Supplementary Material. This reference is also available online at: https://raw.githubusercontent.com/PlantPhenoOntology/ppo/master/documentation/ppo.pdf, and the online version will be updated to reflect changes to the PPO.

Our efforts to merge different phenology data sets using the PPO revealed several challenges of data integration that the PPO cannot solve by itself. We discuss three of these challenges. First, even after integration with the PPO, combined phenology data sets might not be suitable for straightforward statistical analyses. For example, in our efforts to merge USA-NPN, NEON, and PEP725 data, we found substantial differences among these data sources and among taxa in data quality, sampling methodology, and temporal resolution. This is evident in **Figure 5A**, for example, where there appears to be more “noise” in the data for North America than in the data for Europe. This might indicate erroneous data, or it could result from accurate observations of late-season flowering events for plants that were not monitored earlier in a growing season. It is clear then, that even though the PPO provides a path forward for integrating disparate phenology data, analyses of combined data sets still require data cleaning and assessments of fitness for use. Careful consideration of the differences among the various data sources will be required to avoid misleading results. As the PPO highlights these differences, it might also foster future efforts to harmonize global phenology monitoring recommendations.

A second challenge with data integration arises from ambiguities in assigning phenological observations to individual plants. The best data sets are explicit about which plant or plants were observed to generate a particular data point; this is most easily accomplished by assigning plants unique identifier strings. In other data sets, it is sometimes unclear whether records pertain to the same or different plants. For instance, if plant identifiers are not used, and a data set includes multiple observations of the same species from the same location, there is no way to know whether these observations are repeat measurements of the same plant or single observations of different plants. Thus, we strongly encourage all phenology data providers and data collectors to also gather information about which plant or plants were observed for each data point. Assigning globally unique identifiers to the plants observed by phenology data collectors is the best strategy to avoid any ambiguity when the data are used by third party researchers.

The third challenge we encountered in our data integration efforts was due to inconsistencies and errors in the supporting information for phenology observations, including localities, dates, and especially scientific names. Taxonomic inconsistency and ambiguity is a well-known source of frustration for biodiversity data aggregators (Goodwin et al., 2015; Zermoglio et al., 2016), and we found this to be true for phenology data as well. Unfortunately, this is not a trivial problem to solve. We encountered numerous records where scientific names included informal annotations such as question marks or delimited lists of potential taxa. In such cases, confidently assigning these records to a single taxon might well be impossible without more information from the original data collector. Here, it is crucial for data providers to do as much as possible to mitigate these problems, since data cleanup is always easier the closer one is to the original source.

The problem domain of the PPO is currently limited to phenology data that derive from direct observations of individual plants (either in the field or in herbaria) or local plant populations and communities. In the future, we would like to expand the PPO to include indirect, larger-scale methods of gathering aggregated plant phenology data, such as phenocams and satellite imagery. Depending on installation details and plant community characteristics, it is sometimes possible to track individual plants from phenocam photographs, in which case the PPO could already be useful. More often, though, phenocam images give a composite view of a plant community. In these cases, spectral components of the images are used to extract summary information about the phenology of the plants in the photographs (Sonnentag et al., 2012). At still larger scales, satellite-based methods completely exclude the possibility of observing individual plants (for now, anyway) and rely entirely on indirect, spectral analysis techniques. Extending the PPO to accommodate these kinds of phenology data will no doubt be challenging, but we see it as a crucial next step toward full integration and fusion of global plant phenology observation data.

Future development efforts aside, the scientific community has already produced millions of readily accessible, direct observation phenology records and digitized herbarium specimens spanning multiple continents, a diverse assemblage of plant species, and a multitude of data sources. We have just begun to tap the scientific potential of these data. A global, integrated phenology data resource would enable new phenology research at the largest scales, and although many challenges remain to achieve this goal, informatics tools for systematic data integration, such as the PPO and related software, will help make it possible. To this end, we welcome feedback and suggestions for how to improve the PPO so that it is compatible with as many phenology data sources, vocabularies, and research applications as possible. Comments may be left at the PPO issue tracker on GitHub at https://github.com/PlantPhenoOntology/ppo/issues. Our hope is that the PPO, in tandem with ongoing work to build new tools for accessing and aggregating phenology data, will help usher in a new era of global plant phenology research.

## Author Contributions

RW, RG, JD, EGD, and KB conceived of the project and planned the initial community workshop. BJS was the lead ontology developer with all authors contributing to ontology design, evaluation, and testing. JD was the lead developer for the data integration pipeline. BJS wrote the first draft of the manuscript, created the figures, and performed the data analyses. All authors contributed to manuscript revision and approved the submitted version.

## Conflict of Interest Statement

The authors declare that the research was conducted in the absence of any commercial or financial relationships that could be construed as a potential conflict of interest.

## Footnotes

1 https://www.usanpn.org/

2 http://data.neonscience.org/

3 http://www.pep725.eu

4 https://www.naturewatch.ca/plantwatch/

5 http://www.seasonwatch.in/

6 http://www.climatewatch.org.au/

7 https://github.com/stuckyb/ontopilot/

8 https://www.elastic.co/

9 https://www.plantphenology.org/

10 http://data.usanpn.org/observations/

11 http://purl.obolibrary.org/obo/pato.owl

12 http://purl.obolibrary.org/obo/iao.owl

13 http://purl.obolibrary.org/obo/pco.owl
