# Supplementary material for: The Plant Phenology Ontology: A New Informatics Resource for Large-Scale Integration of Plant Phenology Data
Source: Front Plant Sci. 2018 May 1;9:517. doi: 10.3389/fpls.2018.00517 (PMC5938398; doi:10.3389/fpls.2018.00517)
Supplement: Supplementary file 1 [file Data_Sheet_1.pdf]

The Plant Phenology Ontology: Term Definitions and Documentation

This documentation corresponds with the October 20, 2017 release of the PPO (https://raw.githubusercontent.com/PlantPhenoOntology/ppo/master/releases/2017-10-20/ppo.owl) and is supplementary material for: Stucky et al. (2018) The Plant Phenology Ontology: A new informatics resource for large-scale integration of plant phenology data. *Frontiers in Plant Science*. doi: 10.3389/fpls.2018.00517. The most recent version of this documentation is available at https://raw.githubusercontent.com/PlantPhenoOntology/ppo/master/documentation/ppo.pdf.

Contents

1. Introduction 1
2. Phenological traits: top-level trait classes 1
3. Phenological traits: "present" trait classes 12
4. Phenological traits: "absent" trait classes 25
5. Plant structures 66
6. Data properties 83

Introduction

The PPO's model of plant phenology data is based on observations of "phenological traits". Phenological traits are observable features of a plant that provide phenologically relevant information, such as whether a plant has leaves, how many ripe fruits are on a plant, or whether a plant's leaves are senescing. Definitions of phenological traits therefore depend on classes for particular plant structures. The PPO defines a large suite of phenological trait classes for representing information about plant phenology.

The PPO also provides four data properties that are used to record quantitative information about phenological traits. The PPO's trait classes, data properties, and supporting classes from other key ontologies work together to describe the phenology of a particular plant or plant community at some place and time.

Phenological traits: top-level trait classes

Note that each phenological trait "presence" class documented in this section also has two convenience subclasses, one for the case where a particular feature of a plant is present and one for when it is absent. So, for example, 'mature leaf presence' has subclasses 'mature

leaves present' and 'mature leaves absent'. For ease of readibility, these present/absent classes are not included in this section. Instead, they are documented in the sections "Phenological traits: "present" trait classes" and "Phenological traits: "absent" trait classes".

> ### plant structure presence
>
> OBO ID: PPO:0002001
>
> IRI: http://purl.obolibrary.org/obo/PPO_0002001
>
> Definition: A 'plant phenological trait' (PPO:0002000) that is measured by the number of some 'plant structure' (PO:0009011) that are part of a 'whole plant' (PO:0000003).
>
> Comment: The number can be 0 or more.
>
> > #### breaking leaf bud presence
> >
> > OBO ID: PPO:0002013
> >
> > IRI: http://purl.obolibrary.org/obo/PPO_0002013
> >
> > Definition: A 'plant phenological trait' (PPO:0002000) that is measured by the number of breaking leaf buds (PPO:0001012) on a 'whole plant' (PO:0000003).
> >
> > Comment: The number can be 0 or more.
> >
> > #### cone presence
> >
> > OBO ID: PPO:0002048
> >
> > IRI: http://purl.obolibrary.org/obo/PPO_0002048
> >
> > Definition: A 'plant phenological trait' (PPO:0002000) that is measured by the number of cones (strobili) (PO:0025083) on a 'whole plant' (PO:0000003).
> >
> > Comment: The number can be 0 or more.
> >
> > #### dormant leaf bud presence
> >
> > OBO ID: PPO:0002010
> >
> > IRI: http://purl.obolibrary.org/obo/PPO_0002010
> >
> > Definition: A 'plant phenological trait' (PPO:0002000) that is measured by the number of dormant leaf buds (PPO:0001009) on a 'whole plant' (PO:0000003).
> >
> > Comment: The number can be 0 or more.
> >
> > #### expanded immature true leaf presence
> >
> > OBO ID: PPO:0002023
> >
> > IRI: http://purl.obolibrary.org/obo/PPO_0002023
> >
> > Definition: A 'plant phenological trait' (PPO:0002000) that is measured by the number of expanded immature true leaves (PPO:0001021) on a 'whole plant' (PO:0000003).
> >
> > Comment: The number can be 0 or more.
> >
> > #### expanding true leaf presence
> >
> > OBO ID: PPO:0002024

IRI: http://purl.obolibrary.org/obo/PPO_0002024

Definition: A 'plant phenological trait' (PPO:0002000) that is measured by the number of expanding true leaves (PPO:0001022) on a 'whole plant' (PO:0000003).

Comment: The number can be 0 or more.

expanding unfolded true leaf presence

OBO ID: PPO:0002022

IRI: http://purl.obolibrary.org/obo/PPO_0002022

Definition: A 'plant phenological trait' (PPO:0002000) that is measured by the number of expanding unfolded true leaves (PPO:0001020) on a 'whole plant' (PO:0000003).

Comment: The number can be 0 or more.

floral structure presence

OBO ID: PPO:0002026

IRI: http://purl.obolibrary.org/obo/PPO_0002026

Definition: A 'plant phenological trait' (PPO:0002000) that is measured by the number of floral structures (PPO:0001025) on a 'whole plant' (PO:0000003).

Comment: The number can be 0 or more.

flower head presence

OBO ID: PPO:0002038

IRI: http://purl.obolibrary.org/obo/PPO_0002038

Definition: A 'plant phenological trait' (PPO:0002000) that is measured by the number of inflorescences (PO:0009049) on a 'whole plant' (PO:0000003).

Comment: The number can be 0 or more.

flower presence

OBO ID: PPO:0002032

IRI: http://purl.obolibrary.org/obo/PPO_0002032

Definition: A 'plant phenological trait' (PPO:0002000) that is measured by the number of flowers (PO:0009046) on a 'whole plant' (PO:0000003).

Comment: The number can be 0 or more.

fresh pollen cone presence

OBO ID: PPO:0002050

IRI: http://purl.obolibrary.org/obo/PPO_0002050

Definition: A 'plant phenological trait' (PPO:0002000) that is measured by the number of fresh pollen cones (PPO:0001046) on a 'whole plant' (PO:0000003).

Comment: The number can be 0 or more.

fresh seed cone presence

OBO ID: PPO:0002054

IRI: http://purl.obolibrary.org/obo/PPO_0002054

Definition: A 'plant phenological trait' (PPO:0002000) that is measured by the number of fresh seed cones (PPO:0001049) on a 'whole plant' (PO:0000003).

Comment: The number can be 0 or more.

fruit presence

OBO ID: PPO:0002044

IRI: http://purl.obolibrary.org/obo/PPO_0002044

Definition: A 'plant phenological trait' (PPO:0002000) that is measured by the number of simple fruit or compound fruits (PPO:0001042) on a 'whole plant' (PO:0000003).

Comment: The number can be 0 or more.

immature unfolded true leaf presence

OBO ID: PPO:0002020

IRI: http://purl.obolibrary.org/obo/PPO_0002020

Definition: A 'plant phenological trait' (PPO:0002000) that is measured by the number of immature unfolded true leaves (PPO:0001018) on a 'whole plant' (PO:0000003).

Comment: The number can be 0 or more.

leaf bud presence

OBO ID: PPO:0002009

IRI: http://purl.obolibrary.org/obo/PPO_0002009

Definition: A 'plant phenological trait' (PPO:0002000) that is measured by the number of vegetative buds (PO:0000058) on a 'whole plant' (PO:0000003).

Comment: The number can be 0 or more.

mature true leaf presence

OBO ID: PPO:0002021

IRI: http://purl.obolibrary.org/obo/PPO_0002021

Definition: A 'plant phenological trait' (PPO:0002000) that is measured by the number of mature true leaves (PPO:0001019) on a 'whole plant' (PO:0000003).

Comment: The number can be 0 or more.

new above-ground shoot-borne shoot system presence

OBO ID: PPO:0002004

IRI: http://purl.obolibrary.org/obo/PPO_0002004

Definition: A 'plant phenological trait' (PPO:0002000) that is measured by the number of new above-ground shoot-borne shoot systems (PPO:0001004) on a 'whole plant' (PO:0000003).

Comment: The number can be 0 or more.

new shoot system emerging from ground in first growth cycle presence

OBO ID: PPO:0002006

IRI: http://purl.obolibrary.org/obo/PPO_0002006

Definition: A 'plant phenological trait' (PPO:0002000) that is measured by the number of new shoot systems emerging from ground in first growth cycle (PPO:0001006) on a 'whole plant' (PO:0000003).

Comment: The number can be 0 or more.

new shoot system emerging from ground in later growth cycle presence

OBO ID: PPO:0002008

IRI: http://purl.obolibrary.org/obo/PPO_0002008

Definition: A 'plant phenological trait' (PPO:0002000) that is measured by the number of new shoot systems emerging from ground in later growth cycle (PPO:0001008) on a 'whole plant' (PO:0000003).

Comment: The number can be 0 or more.

new shoot system emerging from ground presence

OBO ID: PPO:0002005

IRI: http://purl.obolibrary.org/obo/PPO_0002005

Definition: A 'plant phenological trait' (PPO:0002000) that is measured by the number of new shoot systems emerging from ground (PPO:0001005) on a 'whole plant' (PO:0000003).

Comment: The number can be 0 or more.

new shoot system presence

OBO ID: PPO:0002003

IRI: http://purl.obolibrary.org/obo/PPO_0002003

Definition: A 'plant phenological trait' (PPO:0002000) that is measured by the number of new shoot systems (PPO:0001003) on a 'whole plant' (PO:0000003).

Comment: The number can be 0 or more.

non-dormant leaf bud presence

OBO ID: PPO:0002011

IRI: http://purl.obolibrary.org/obo/PPO_0002011

Definition: A 'plant phenological trait' (PPO:0002000) that is measured by the number of non-dormant leaf buds (PPO:0001010) on a 'whole plant' (PO:0000003).

Comment: The number can be 0 or more.

non-senesced floral structure presence

OBO ID: PPO:0002027

IRI: http://purl.obolibrary.org/obo/PPO_0002027

Definition: A 'plant phenological trait' (PPO:0002000) that is measured by the number of non-senesced floral structures (PPO:0001026) on a 'whole plant' (PO:0000003).

Comment: The number can be 0 or more.

non-senesced flower head presence

OBO ID: PPO:0002039

IRI: http://purl.obolibrary.org/obo/PPO_0002039

Definition: A 'plant phenological trait' (PPO:0002000) that is measured by the number of non-senesced flower heads (PPO:0001036) on a 'whole plant' (PO:0000003).

Comment: The number can be 0 or more.

non-senesced flower presence

OBO ID: PPO:0002033

IRI: http://purl.obolibrary.org/obo/PPO_0002033

Definition: A 'plant phenological trait' (PPO:0002000) that is measured by the number of non-senesced flowers (PPO:0001031) on a 'whole plant' (PO:0000003).

Comment: The number can be 0 or more.

non-senescing unfolded true leaf presence

OBO ID: PPO:0002018

IRI: http://purl.obolibrary.org/obo/PPO_0002018

Definition: A 'plant phenological trait' (PPO:0002000) that is measured by the number of non-senescing unfolded true leaves (PPO:0001016) on a 'whole plant' (PO:0000003).

Comment: The number can be 0 or more.

open floral structure presence

OBO ID: PPO:0002029

IRI: http://purl.obolibrary.org/obo/PPO_0002029

Definition: A 'plant phenological trait' (PPO:0002000) that is measured by the number of open floral structures (PPO:0001028) on a 'whole plant' (PO:0000003).

Comment: The number can be 0 or more.

open flower head presence

OBO ID: PPO:0002041

IRI: http://purl.obolibrary.org/obo/PPO_0002041

Definition: A 'plant phenological trait' (PPO:0002000) that is measured by the number of open flower heads (PPO:0001038) on a 'whole plant' (PO:0000003).

Comment: The number can be 0 or more.

open flower presence

OBO ID: PPO:0002035

IRI: http://purl.obolibrary.org/obo/PPO_0002035

Definition: A 'plant phenological trait' (PPO:0002000) that is measured by the number of open flowers (PPO:0001033) on a 'whole plant' (PO:0000003).

Comment: The number can be 0 or more.

open pollen cone presence

OBO ID: PPO:0002051

IRI: http://purl.obolibrary.org/obo/PPO_0002051

Definition: A 'plant phenological trait' (PPO:0002000) that is measured by the number of open pollen cones (PPO:0001047) on a 'whole plant' (PO:0000003).

Comment: The number can be 0 or more.

pollen cone presence

OBO ID: PPO:0002049

IRI: http://purl.obolibrary.org/obo/PPO_0002049

Definition: A 'plant phenological trait' (PPO:0002000) that is measured by the number of pollen cones (PO:0005031) on a 'whole plant' (PO:0000003).

Comment: The number can be 0 or more.

pollen-releasing floral structure presence

OBO ID: PPO:0002030

IRI: http://purl.obolibrary.org/obo/PPO_0002030

Definition: A 'plant phenological trait' (PPO:0002000) that is measured by the number of pollen-releasing floral structures (PPO:0001029) on a 'whole plant' (PO:0000003).

Comment: The number can be 0 or more.

pollen-releasing flower head presence

OBO ID: PPO:0002042

IRI: http://purl.obolibrary.org/obo/PPO_0002042

Definition: A 'plant phenological trait' (PPO:0002000) that is measured by the number of pollen-releasing flower heads (PPO:0001039) on a 'whole plant' (PO:0000003).

Comment: The number can be 0 or more.

pollen-releasing flower presence

OBO ID: PPO:0002036

IRI: http://purl.obolibrary.org/obo/PPO_0002036

Definition: A 'plant phenological trait' (PPO:0002000) that is measured by the number of pollen-releasing flowers (PPO:0001034) on a 'whole plant' (PO:0000003).

Comment: The number can be 0 or more.

pollen-releasing pollen cone presence

OBO ID: PPO:0002052

IRI: http://purl.obolibrary.org/obo/PPO_0002052

Definition: A 'plant phenological trait' (PPO:0002000) that is measured by the number of pollen-releasing pollen cones (PPO:0001048) on a 'whole plant' (PO:0000003).

Comment: The number can be 0 or more.

reproductive structure presence

OBO ID: PPO:0002025

IRI: http://purl.obolibrary.org/obo/PPO_0002025

Definition: A 'plant phenological trait' (PPO:0002000) that is measured by the number of reproductive structures (PPO:0001023) on a 'whole plant' (PO:0000003).

Comment: The number can be 0 or more.

ripe fruit presence

OBO ID: PPO:0002047

IRI: http://purl.obolibrary.org/obo/PPO_0002047

Definition: A 'plant phenological trait' (PPO:0002000) that is measured by the number of ripe fruits (PPO:0001045) on a 'whole plant' (PO:0000003).

Comment: The number can be 0 or more.

ripe seed cone presence

OBO ID: PPO:0002057

IRI: http://purl.obolibrary.org/obo/PPO_0002057

Definition: A 'plant phenological trait' (PPO:0002000) that is measured by the number of ripe seed cones (PPO:0001052) on a 'whole plant' (PO:0000003).

Comment: The number can be 0 or more.

ripening fruit presence

OBO ID: PPO:0002045

IRI: http://purl.obolibrary.org/obo/PPO_0002045

Definition: A 'plant phenological trait' (PPO:0002000) that is measured by the number of ripening fruits (PPO:0001043) on a 'whole plant' (PO:0000003).

Comment: The number can be 0 or more.

ripening seed cone presence

OBO ID: PPO:0002055

IRI: http://purl.obolibrary.org/obo/PPO_0002055

Definition: A 'plant phenological trait' (PPO:0002000) that is measured by the number of ripening seed cones (PPO:0001050) on a 'whole plant' (PO:0000003).

Comment: The number can be 0 or more.

seed cone presence

OBO ID: PPO:0002053

IRI: http://purl.obolibrary.org/obo/PPO_0002053

Definition: A 'plant phenological trait' (PPO:0002000) that is measured by the number of seed cones (PO:0005032) on a 'whole plant' (PO:0000003).

Comment: The number can be 0 or more.

seedling presence

OBO ID: PPO:0002007

IRI: http://purl.obolibrary.org/obo/PPO_0002007

Definition: A 'plant phenological trait' (PPO:0002000) that is measured by whether or not a 'whole plant' (PO:0000003) is a 'seedling' (PPO:0001007).

Comment: For consistency with other trait measurements, 'seedling presence' is also measured with a count, which must be a binary value: 0 if the plant is a seedling, 1 if it is not.

senesced floral structure presence

OBO ID: PPO:0002031

IRI: http://purl.obolibrary.org/obo/PPO_0002031

Definition: A 'plant phenological trait' (PPO:0002000) that is measured by the number of senesced floral structures (PPO:0001030) on a 'whole plant' (PO:0000003).

Comment: The number can be 0 or more.

senesced flower head presence

OBO ID: PPO:0002043

IRI: http://purl.obolibrary.org/obo/PPO_0002043

Definition: A 'plant phenological trait' (PPO:0002000) that is measured by the number of senesced flower heads (PPO:0001040) on a 'whole plant' (PO:0000003).

Comment: The number can be 0 or more.

senesced flower presence

OBO ID: PPO:0002037

IRI: http://purl.obolibrary.org/obo/PPO_0002037

Definition: A 'plant phenological trait' (PPO:0002000) that is measured by the number of senesced flowers (PPO:0001035) on a 'whole plant' (PO:0000003).

Comment: The number can be 0 or more.

senescing true leaf presence

OBO ID: PPO:0002019

IRI: http://purl.obolibrary.org/obo/PPO_0002019

Definition: A 'plant phenological trait' (PPO:0002000) that is measured by the number of senescing true leaves (PPO:0001017) on a 'whole plant' (PO:0000003).

Comment: The number can be 0 or more.

swelling leaf bud presence

OBO ID: PPO:0002012

IRI: http://purl.obolibrary.org/obo/PPO_0002012

Definition: A 'plant phenological trait' (PPO:0002000) that is measured by the number of swelling leaf buds (PPO:0001011) on a 'whole plant' (PO:0000003).

Comment: The number can be 0 or more.

true leaf presence

OBO ID: PPO:0002015

IRI: http://purl.obolibrary.org/obo/PPO_0002015

Definition: A 'plant phenological trait' (PPO:0002000) that is measured by the number of true leaves (PPO:0001013) on a 'whole plant' (PO:0000003).

Comment: The number can be 0 or more.

unfolded true leaf presence

OBO ID: PPO:0002017

IRI: http://purl.obolibrary.org/obo/PPO_0002017

Definition: A 'plant phenological trait' (PPO:0002000) that is measured by the number of unfolded true leaves (PPO:0001015) on a 'whole plant' (PO:0000003).

Comment: The number can be 0 or more.

unfolding true leaf presence

OBO ID: PPO:0002016

IRI: http://purl.obolibrary.org/obo/PPO_0002016

Definition: A 'plant phenological trait' (PPO:0002000) that is measured by the number of unfolding true leaves (PPO:0001014) on a 'whole plant' (PO:0000003).

Comment: The number can be 0 or more.

unopened floral structure presence

OBO ID: PPO:0002028

IRI: http://purl.obolibrary.org/obo/PPO_0002028

Definition: A 'plant phenological trait' (PPO:0002000) that is measured by the number of unopened floral structures (PPO:0001027) on a 'whole plant' (PO:0000003).

Comment: The number can be 0 or more.

unopened flower head presence

OBO ID: PPO:0002040

IRI: http://purl.obolibrary.org/obo/PPO_0002040

Definition: A 'plant phenological trait' (PPO:0002000) that is measured by the number of unopened flower heads (PPO:0001037) on a 'whole plant' (PO:0000003).

Comment: The number can be 0 or more.

unopened flower presence

OBO ID: PPO:0002034

IRI: http://purl.obolibrary.org/obo/PPO_0002034

Definition: A 'plant phenological trait' (PPO:0002000) that is measured by the number of unopened flowers (PPO:0001032) on a 'whole plant' (PO:0000003).

Comment: The number can be 0 or more.

unripe fruit presence

OBO ID: PPO:0002046

IRI: http://purl.obolibrary.org/obo/PPO_0002046

Definition: A 'plant phenological trait' (PPO:0002000) that is measured by the number of unripe fruits (PPO:0001044) on a 'whole plant' (PO:0000003).

Comment: The number can be 0 or more.

unripe seed cone presence

OBO ID: PPO:0002056

IRI: http://purl.obolibrary.org/obo/PPO_0002056

Definition: A 'plant phenological trait' (PPO:0002000) that is measured by the number of unripe seed cones (PPO:0001051) on a 'whole plant' (PO:0000003).

Comment: The number can be 0 or more.

vascular leaf presence

OBO ID: PPO:0002014

IRI: http://purl.obolibrary.org/obo/PPO_0002014

Definition: A 'plant phenological trait' (PPO:0002000) that is measured by the number of vascular leaves (PO:0009025) on a 'whole plant' (PO:0000003).

Comment: The number can be 0 or more.

abscised plant structure presence

OBO ID: PPO:0002002

IRI: http://purl.obolibrary.org/obo/PPO_0002002

Definition: A 'plant phenological trait' (PPO:0002000) that is measured by the number of some 'plant structure' (PO:0009011) that a 'whole plant' (PO:0000003) has abscised or that have been removed from a 'whole plant' (PO:0000003).

Comment: The number can be 0 or more.

abscised cone or seed presence

OBO ID: PPO:0002060

IRI: http://purl.obolibrary.org/obo/PPO_0002060

Definition: A 'plant phenological trait' (PPO:0002000) that is measured by the number of a whole plant's ripe seed cones (PPO:0001052) that have been abscised or removed by an herbivore and/or the number of ripe seed cones on a 'whole plant' (PO:0000003) that have abscised at least one 'mature seed' (PPO:0001024).

Comment: The number can be 0 or more and only includes the most recent cone crop.

abscised fruit or seed presence

OBO ID: PPO:0002059

IRI: http://purl.obolibrary.org/obo/PPO_0002059

Definition: A 'plant phenological trait' (PPO:0002000) that is measured by the number of a whole plant's ripe fruits (PPO:0001045) that have been abscised or removed by an herbivore and/or the number of ripe fruits on a 'whole plant' (PO:0000003) that have abscised at least one 'mature seed' (PPO:0001024).

Comment: The number can be 0 or more.

abscised leaf presence

OBO ID: PPO:0002058

IRI: http://purl.obolibrary.org/obo/PPO_0002058

Definition: A 'plant phenological trait' (PPO:0002000) that is measured by the number of true leaves (PPO:0001013) that a 'whole plant' (PO:0000003) has abscised.

Comment: The number can be 0 or more.

Phenological traits: "present" trait classes

new shoot system present

OBO ID: PPO:0002301

IRI: http://purl.obolibrary.org/obo/PPO_0002301

Definition: An 'new shoot system presence' (PPO:0002003) trait that is a 'quality of' (RO:0000080) a 'whole plant' (PO:0000003) that has at least one 'new shoot system' (PPO:0001003).

new above-ground shoot-borne shoot systems present

OBO ID: PPO:0002302

IRI: http://purl.obolibrary.org/obo/PPO_0002302

Definition: A 'new above-ground shoot-borne shoot system presence' (PPO:0002004) trait that is a 'quality of' (RO:0000080) a 'whole plant' (PO:0000003) that has at least one 'new above-ground shoot-borne shoot system' (PPO:0001004).

breaking leaf buds present

OBO ID: PPO:0002311

IRI: http://purl.obolibrary.org/obo/PPO_0002311

Definition: A 'breaking leaf bud presence' (PPO:0002013) trait that is a 'quality of' (RO:0000080) a 'whole plant' (PO:0000003) that has at least one 'breaking leaf bud' (PPO:0001012).

new shoot systems emerging from ground present

OBO ID: PPO:0002303

IRI: http://purl.obolibrary.org/obo/PPO_0002303

Definition: A 'new shoot system emerging from ground presence' (PPO:0002005) trait that is a 'quality of' (RO:0000080) a 'whole plant' (PO:0000003) that has at least one 'new shoot system emerging from ground' (PPO:0001005).

new shoot systems emerging from ground in first growth cycle present

OBO ID: PPO:0002304

IRI: http://purl.obolibrary.org/obo/PPO_0002304

Definition: A 'new shoot system emerging from ground in first growth cycle presence' (PPO:0002006) trait that is a 'quality of' (RO:0000080) a 'whole plant' (PO:0000003) that has at least one 'new shoot system emerging from ground in first growth cycle' (PPO:0001006).

seedling present

OBO ID: PPO:0002305

IRI: http://purl.obolibrary.org/obo/PPO_0002305

Definition: A 'seedling presence' (PPO:0002007) trait that is a 'quality of' (RO:0000080) a 'whole plant' (PO:0000003) that is a 'seedling' (PPO:0001007).

new shoot systems emerging from ground in later growth cycle present

OBO ID: PPO:0002306

IRI: http://purl.obolibrary.org/obo/PPO_0002306

Definition: A 'new shoot system emerging from ground in later growth cycle presence' (PPO:0002008) trait that is a 'quality of' (RO:0000080) a 'whole plant' (PO:0000003) that has at least one 'new shoot system emerging from ground in later growth cycle' (PPO:0001008).

leaf buds present

OBO ID: PPO:0002307

IRI: http://purl.obolibrary.org/obo/PPO_0002307

Definition: A 'leaf bud presence' (PPO:0002009) trait that is a 'quality of' (RO:0000080) a 'whole plant' (PO:0000003) that has at least one PO:'vegetative bud' (PO:0000058).

dormant leaf buds present

OBO ID: PPO:0002308

IRI: http://purl.obolibrary.org/obo/PPO_0002308

Definition: A 'dormant leaf bud presence' (PPO:0002010) trait that is a 'quality of' (RO:0000080) a 'whole plant' (PO:0000003) that has at least one 'dormant leaf bud' (PPO:0001009).

non-dormant leaf buds present

OBO ID: PPO:0002309

IRI: http://purl.obolibrary.org/obo/PPO_0002309

Definition: A 'non-dormant leaf bud presence' (PPO:0002011) trait that is a 'quality of' (RO:0000080) a 'whole plant' (PO:0000003) that has at least one 'non-dormant leaf bud' (PPO:0001010).

breaking leaf buds present

OBO ID: PPO:0002311

IRI: http://purl.obolibrary.org/obo/PPO_0002311

Definition: A 'breaking leaf bud presence' (PPO:0002013) trait that is a 'quality of' (RO:0000080) a 'whole plant' (PO:0000003) that has at least one 'breaking leaf bud' (PPO:0001012).

swelling leaf buds present

OBO ID: PPO:0002310

IRI: http://purl.obolibrary.org/obo/PPO_0002310

Definition: A 'swelling leaf bud presence' (PPO:0002012) trait that is a 'quality of' (RO:0000080) a 'whole plant' (PO:0000003) that has at least one 'swelling leaf bud' (PPO:0001011).

vascular leaves present

OBO ID: PPO:0002312

IRI: http://purl.obolibrary.org/obo/PPO_0002312

Definition: A 'vascular leaf presence' (PPO:0002014) trait that is a 'quality of' (RO:0000080) a 'whole plant' (PO:0000003) that has at least one 'vascular leaf' (PO:0009025).

true leaves present

OBO ID: PPO:0002313

IRI: http://purl.obolibrary.org/obo/PPO_0002313

Definition: A 'true leaf presence' (PPO:0002015) trait that is a 'quality of' (RO:0000080) a 'whole plant' (PO:0000003) that has at least one 'true leaf' (PPO:0001013).

expanding true leaves present

OBO ID: PPO:0002322

IRI: http://purl.obolibrary.org/obo/PPO_0002322

Definition: An 'expanding true leaf presence' (PPO:0002024) trait that is a 'quality of' (RO:0000080) a 'whole plant' (PO:0000003) that has at least one 'expanding true leaf' (PPO:0001022).

expanding unfolded true leaves present

OBO ID: PPO:0002320

IRI: http://purl.obolibrary.org/obo/PPO_0002320

Definition: An 'expanding unfolded true leaf presence' (PPO:0002022) trait that is a 'quality of' (RO:0000080) a 'whole plant' (PO:0000003) that has at least one 'expanding unfolded true leaf' (PPO:0001020).

unfolding true leaves present

OBO ID: PPO:0002314

IRI: http://purl.obolibrary.org/obo/PPO_0002314

Definition: An 'unfolding true leaf presence' (PPO:0002016) trait that is a 'quality of' (RO:0000080) a 'whole plant' (PO:0000003) that has at least one 'unfolding true leaf' (PPO:0001014).

breaking leaf buds present

OBO ID: PPO:0002311

IRI: http://purl.obolibrary.org/obo/PPO_0002311

Definition: A 'breaking leaf bud presence' (PPO:0002013) trait that is a 'quality of' (RO:0000080) a 'whole plant' (PO:0000003) that has at least one 'breaking leaf bud' (PPO:0001012).

unfolded true leaves present

OBO ID: PPO:0002315

IRI: http://purl.obolibrary.org/obo/PPO_0002315

Definition: An 'unfolded true leaf presence' (PPO:0002017) trait that is a 'quality of' (RO:0000080) a 'whole plant' (PO:0000003) that has at least one 'unfolded true leaf' (PPO:0001015).

non-senescing unfolded true leaves present

OBO ID: PPO:0002316

IRI: http://purl.obolibrary.org/obo/PPO_0002316

Definition: A 'non-senescing unfolded true leaf presence' (PPO:0002018) trait that is a 'quality of' (RO:0000080) a 'whole plant' (PO:0000003) that has at least one 'non-senescing unfolded true leaf' (PPO:0001016).

immature unfolded true leaves present

OBO ID: PPO:0002318

IRI: http://purl.obolibrary.org/obo/PPO_0002318

Definition: An 'immature unfolded true leaf presence' (PPO:0002020) trait that is a 'quality of' (RO:0000080) a 'whole plant' (PO:0000003) that has at least one 'immature unfolded true leaf' (PPO:0001018).

expanded immature true leaves present

OBO ID: PPO:0002321

IRI: http://purl.obolibrary.org/obo/PPO_0002321

Definition: An 'expanded immature true leaf presence' (PPO:0002023) trait that is a 'quality of' (RO:0000080) a 'whole plant' (PO:0000003) that has at least one 'expanded immature true leaf' (PPO:0001021).

expanding unfolded true leaves present

OBO ID: PPO:0002320

IRI: http://purl.obolibrary.org/obo/PPO_0002320

Definition: An 'expanding unfolded true leaf presence' (PPO:0002022) trait that is a 'quality of' (RO:0000080) a 'whole plant' (PO:0000003) that has at least one 'expanding unfolded true leaf' (PPO:0001020).

mature true leaves present

OBO ID: PPO:0002319

IRI: http://purl.obolibrary.org/obo/PPO_0002319

Definition: An 'mature true leaf presence' (PPO:0002021) trait that is a 'quality of' (RO:0000080) a 'whole plant' (PO:0000003) that has at least one 'mature true leaf' (PPO:0001019).

senescing true leaves present

OBO ID: PPO:0002317

IRI: http://purl.obolibrary.org/obo/PPO_0002317

Definition: A 'senescing true leaf presence' (PPO:0002019) trait that is a 'quality of' (RO:0000080) a 'whole plant' (PO:0000003) that has at least one 'senescing true leaf' (PPO:0001017).

reproductive structures present

OBO ID: PPO:0002323

IRI: http://purl.obolibrary.org/obo/PPO_0002323

Definition: A 'reproductive structure presence' (PPO:0002025) trait that is a 'quality of' (RO:0000080) a 'whole plant' (PO:0000003) that has at least one 'reproductive structure' (PPO:0001023).

cones present

OBO ID: PPO:0002346

IRI: http://purl.obolibrary.org/obo/PPO_0002346

Definition: A 'cone presence' (PPO:0002048) trait that is a 'quality of' (RO:0000080) a 'whole plant' (PO:0000003) that has at least one cone (PO:0025083).

pollen cones present

OBO ID: PPO:0002347

IRI: http://purl.obolibrary.org/obo/PPO_0002347

Definition: A 'pollen cone presence' (PPO:0002049) trait that is a 'quality of' (RO:0000080) a 'whole plant' (PO:0000003) that has at least one pollen cone (PO:0005031).

fresh pollen cones present

OBO ID: PPO:0002348

IRI: http://purl.obolibrary.org/obo/PPO_0002348

Definition: A 'fresh pollen cone presence' (PPO:0002050) trait that is a 'quality of' (RO:0000080) a 'whole plant' (PO:0000003) that has at least one 'fresh pollen cone' (PPO:0001046).

open pollen cones present

OBO ID: PPO:0002349

IRI: http://purl.obolibrary.org/obo/PPO_0002349

Definition: An 'open pollen cone presence' (PPO:0002051) trait that is a 'quality of' (RO:0000080) a 'whole plant' (PO:0000003) that has at least one 'open pollen cone' (PPO:0001047).

pollen-releasing pollen cones present

OBO ID: PPO:0002350

IRI: http://purl.obolibrary.org/obo/PPO_0002350

Definition: A 'pollen-releasing pollen cone presence' (PPO:0002052) trait that is a 'quality of' (RO:0000080) a 'whole plant' (PO:0000003) that has at least one 'pollen-releasing pollen cone' (PPO:0001048).

seed cones present

OBO ID: PPO:0002351

IRI: http://purl.obolibrary.org/obo/PPO_0002351

Definition: A 'seed cone presence' (PPO:0002053) trait that is a 'quality of' (RO:0000080) a 'whole plant' (PO:0000003) that has at least one PO:'megasporangiate strobilus' (PO:0005032).

fresh seed cones present

OBO ID: PPO:0002352

IRI: http://purl.obolibrary.org/obo/PPO_0002352

Definition: A 'fresh seed cone presence' (PPO:0002054) trait that is a 'quality of' (RO:0000080) a 'whole plant' (PO:0000003) that has at least one 'fresh seed cone' (PPO:0001049).

ripening seed cones present

OBO ID: PPO:0002353

IRI: http://purl.obolibrary.org/obo/PPO_0002353

Definition: A 'ripening seed cone presence' (PPO:0002055) trait that is a 'quality of' (RO:0000080) a 'whole plant' (PO:0000003) that has at least one 'ripening seed cone' (PPO:0001050).

ripe seed cones present

OBO ID: PPO:0002355

IRI: http://purl.obolibrary.org/obo/PPO_0002355

Definition: A 'ripe seed cone presence' (PPO:0002057) trait that is a 'quality of' (RO:0000080) a 'whole plant' (PO:0000003) that has at least one 'ripe seed cone' (PPO:0001052).

unripe seed cones present

OBO ID: PPO:0002354

IRI: http://purl.obolibrary.org/obo/PPO_0002354

Definition: An 'unripe seed cone presence' (PPO:0002056) trait that is a 'quality of' (RO:0000080) a 'whole plant' (PO:0000003) that has at least one 'unripe seed cone' (PPO:0001051).

floral structures present

OBO ID: PPO:0002324

IRI: http://purl.obolibrary.org/obo/PPO_0002324

Definition: A 'floral structure presence' (PPO:0002026) trait that is a 'quality of' (RO:0000080) a 'whole plant' (PO:0000003) that has at least one 'floral structure' (PPO:0001025).

flower heads present

OBO ID: PPO:0002336

IRI: http://purl.obolibrary.org/obo/PPO_0002336

Definition: A 'flower head presence' (PPO:0002038) trait that is a 'quality of' (RO:0000080) a 'whole plant' (PO:0000003) that has at least one PO:'inflorescence' (PO:0009049).

non-senesced flower heads present

OBO ID: PPO:0002337

IRI: http://purl.obolibrary.org/obo/PPO_0002337

Definition: A 'non-senesced flower head presence' (PPO:0002039) trait that is a 'quality of' (RO:0000080) a 'whole plant' (PO:0000003) that has at least one 'non-senesced flower head' (PPO:0001036).

open flower heads present

OBO ID: PPO:0002339

IRI: http://purl.obolibrary.org/obo/PPO_0002339

Definition: An 'open flower head presence' (PPO:0002041) trait that is a 'quality of' (RO:0000080) a 'whole plant' (PO:0000003) that has at least one 'open flower head' (PPO:0001038).

pollen-releasing flower heads present

OBO ID: PPO:0002340

IRI: http://purl.obolibrary.org/obo/PPO_0002340

Definition: A 'pollen-releasing flower head presence' (PPO:0002042) trait that is a 'quality of' (RO:0000080) a 'whole plant' (PO:0000003) that has at least one 'pollen-releasing flower head' (PPO:0001039).

unopened flower heads present

OBO ID: PPO:0002338

IRI: http://purl.obolibrary.org/obo/PPO_0002338

Definition: An 'unopened flower head presence' (PPO:0002040) trait that is a 'quality of' (RO:0000080) a 'whole plant' (PO:0000003) that has at least one 'unopened flower head' (PPO:0001037).

senesced flower heads present

OBO ID: PPO:0002341

IRI: http://purl.obolibrary.org/obo/PPO_0002341

Definition: A 'senesced flower head presence' (PPO:0002043) trait that is a 'quality of' (RO:0000080) a 'whole plant' (PO:0000003) that has at least one 'senesced flower head' (PPO:0001040).

flowers present

OBO ID: PPO:0002330

IRI: http://purl.obolibrary.org/obo/PPO_0002330

Definition: A 'flower presence' (PPO:0002032) trait that is a 'quality of' (RO:0000080) a 'whole plant' (PO:0000003) that has at least one PO:'flower' (PO:0009046).

non-senesced flowers present

OBO ID: PPO:0002331

IRI: http://purl.obolibrary.org/obo/PPO_0002331

Definition: A 'non-senesced flower presence' (PPO:0002033) trait that is a 'quality of' (RO:0000080) a 'whole plant' (PO:0000003) that has at least one 'non-senesced flower' (PPO:0001031).

open flowers present

OBO ID: PPO:0002333

IRI: http://purl.obolibrary.org/obo/PPO_0002333

Definition: An 'open flower presence' (PPO:0002035) trait that is a 'quality of' (RO:0000080) a 'whole plant' (PO:0000003) that has at least one 'open flower' (PPO:0001033).

open flower heads present

OBO ID: PPO:0002339

IRI: http://purl.obolibrary.org/obo/PPO_0002339

Definition: An 'open flower head presence' (PPO:0002041) trait that is a 'quality of' (RO:0000080) a 'whole plant' (PO:0000003) that has at least one 'open flower head' (PPO:0001038).

pollen-releasing flower heads present

OBO ID: PPO:0002340

IRI: http://purl.obolibrary.org/obo/PPO_0002340

Definition: A 'pollen-releasing flower head presence' (PPO:0002042) trait that is a 'quality of' (RO:0000080) a 'whole plant' (PO:0000003) that has at least one 'pollen-releasing flower head' (PPO:0001039).

pollen-releasing flowers present

OBO ID: PPO:0002334

IRI: http://purl.obolibrary.org/obo/PPO_0002334

Definition: A 'pollen-releasing flower presence' (PPO:0002036) trait that is a 'quality of' (RO:0000080) a 'whole plant' (PO:0000003) that has at least one 'pollen-releasing flower' (PPO:0001034).

pollen-releasing floral structures present

OBO ID: PPO:0002328

IRI: http://purl.obolibrary.org/obo/PPO_0002328

Definition: A 'pollen-releasing floral structure presence' (PPO:0002030) trait that is a 'quality of' (RO:0000080) a 'whole plant' (PO:0000003) that has at least one 'pollen-releasing floral structure' (PPO:0001029).

pollen-releasing flower heads present

OBO ID: PPO:0002340

IRI: http://purl.obolibrary.org/obo/PPO_0002340

Definition: A 'pollen-releasing flower head presence' (PPO:0002042) trait that is a 'quality of' (RO:0000080) a 'whole plant' (PO:0000003) that has at least one 'pollen-releasing flower head' (PPO:0001039).

unopened flowers present

OBO ID: PPO:0002332

IRI: http://purl.obolibrary.org/obo/PPO_0002332

Definition: An 'unopened flower presence' (PPO:0002034) trait that is a 'quality of' (RO:0000080) a 'whole plant' (PO:0000003) that has at least one 'unopened flower' (PPO:0001032).

open floral structures present

OBO ID: PPO:0002327

IRI: http://purl.obolibrary.org/obo/PPO_0002327

Definition: An 'open floral structure presence' (PPO:0002029) trait that is a 'quality of' (RO:0000080) a 'whole plant' (PO:0000003) that has at least one 'open floral structure' (PPO:0001028).

pollen-releasing floral structures present

OBO ID: PPO:0002328

IRI: http://purl.obolibrary.org/obo/PPO_0002328

Definition: A 'pollen-releasing floral structure presence' (PPO:0002030) trait that is a 'quality of' (RO:0000080) a 'whole plant' (PO:0000003) that has at least one 'pollen-releasing floral structure' (PPO:0001029).

senesced flowers present

OBO ID: PPO:0002335

IRI: http://purl.obolibrary.org/obo/PPO_0002335

Definition: A 'senesced flower presence' (PPO:0002037) trait that is a 'quality of' (RO:0000080) a 'whole plant' (PO:0000003) that has at least one 'senesced flower' (PPO:0001035).

senesced floral structures present

OBO ID: PPO:0002329

IRI: http://purl.obolibrary.org/obo/PPO_0002329

Definition: A 'senesced floral structure presence' (PPO:0002031) trait that is a 'quality of' (RO:0000080) a 'whole plant' (PO:0000003) that has at least one 'senesced floral structure' (PPO:0001030).

non-senesced floral structures present

OBO ID: PPO:0002325

IRI: http://purl.obolibrary.org/obo/PPO_0002325

Definition: A 'non-senesced floral structure presence' (PPO:0002027) trait that is a 'quality of' (RO:0000080) a 'whole plant' (PO:0000003) that has at least one 'non-senesced floral structure' (PPO:0001026).

open floral structures present

OBO ID: PPO:0002327

IRI: http://purl.obolibrary.org/obo/PPO_0002327

Definition: An 'open floral structure presence' (PPO:0002029) trait that is a 'quality of' (RO:0000080) a 'whole plant' (PO:0000003) that has at least one 'open floral structure' (PPO:0001028).

pollen-releasing floral structures present

OBO ID: PPO:0002328

IRI: http://purl.obolibrary.org/obo/PPO_0002328

Definition: A 'pollen-releasing floral structure presence' (PPO:0002030) trait that is a 'quality of' (RO:0000080) a 'whole plant' (PO:0000003) that has at least one 'pollen-releasing floral structure' (PPO:0001029).

unopened floral structures present

OBO ID: PPO:0002326

IRI: http://purl.obolibrary.org/obo/PPO_0002326

Definition: An 'unopened floral structure presence' (PPO:0002028) trait that is a 'quality of' (RO:0000080) a 'whole plant' (PO:0000003) that has at least one 'unopened floral structure' (PPO:0001027).

fruits present

OBO ID: PPO:0002342

IRI: http://purl.obolibrary.org/obo/PPO_0002342

Definition: A 'fruit presence' (PPO:0002044) trait that is a 'quality of' (RO:0000080) a 'whole plant' (PO:0000003) that has at least one 'simple fruit or compound fruit' (PPO:0001042).

ripening fruits present

OBO ID: PPO:0002343

IRI: http://purl.obolibrary.org/obo/PPO_0002343

Definition: A 'ripening fruit presence' (PPO:0002045) trait that is a 'quality of' (RO:0000080) a 'whole plant' (PO:0000003) that has at least one 'ripening fruit' (PPO:0001043).

ripe fruits present

OBO ID: PPO:0002345

IRI: http://purl.obolibrary.org/obo/PPO_0002345

Definition: A 'ripe fruit presence' (PPO:0002047) trait that is a 'quality of' (RO:0000080) a 'whole plant' (PO:0000003) that has at least one 'ripe fruit' (PPO:0001045).

unripe fruits present

OBO ID: PPO:0002344

IRI: http://purl.obolibrary.org/obo/PPO_0002344

Definition: An 'unripe fruit presence' (PPO:0002046) trait that is a 'quality of' (RO:0000080) a 'whole plant' (PO:0000003) that has at least one 'unripe fruit' (PPO:0001044).

abscised plant structures present

OBO ID: PPO:0002356

IRI: http://purl.obolibrary.org/obo/PPO_0002356

Definition: An 'abscised plant structure presence' (PPO:0002002) trait that is a 'quality of' (RO:0000080) a 'whole plant' (PO:0000003) from which at least one of some 'plant structure' (PO:0009011) has been abscised or removed.

abscised cones or seeds present

OBO ID: PPO:0002359

IRI: http://purl.obolibrary.org/obo/PPO_0002359

Definition: An 'abscised cone or seed presence' (PPO:0002060) trait that is a 'quality of' (RO:0000080) a 'whole plant' (PO:0000003) from which at least one 'ripe seed cone' (PPO:0001052) has been abscised or removed by an herbivore or that has at least one ripe seed cone that has 'abscised' (PPO:0000005) at least one 'mature seed' (PPO:0001024).

abscised fruits or seeds present

OBO ID: PPO:0002358

IRI: http://purl.obolibrary.org/obo/PPO_0002358

Definition: An 'abscised fruit or seed presence' (PPO:0002059) trait that is a 'quality of' (RO:0000080) a 'whole plant' (PO:0000003) from which at least one 'ripe fruit' (PPO:0001045) has been abscised or removed by an herbivore or that has at least one 'ripe fruit' (PPO:0001045) that has abscised at least one 'mature seed' (PPO:0001024).

abscised leaves present

OBO ID: PPO:0002357

IRI: http://purl.obolibrary.org/obo/PPO_0002357

Definition: An 'abscised leaf presence' (PPO:0002058) trait that is a 'quality of' (RO:0000080) a 'whole plant' (PO:0000003) from which at least one 'true leaf' (PPO:0001013) has been 'abscised' (PPO:0000005).

Phenological traits: "absent" trait classes

breaking leaf buds absent

OBO ID: PPO:0002610

IRI: http://purl.obolibrary.org/obo/PPO_0002610

Definition: A 'breaking leaf bud presence' (PPO:0002013) trait that is a 'quality of' (RO:0000080) a 'whole plant' (PO:0000003) that does not have any breaking leaf buds (PPO:0001012).

new above-ground shoot-borne shoot systems absent

OBO ID: PPO:0002601

IRI: http://purl.obolibrary.org/obo/PPO_0002601

Definition: A 'new above-ground shoot-borne shoot system presence' (PPO:0002004) trait that is a 'quality of' (RO:0000080) a 'whole plant' (PO:0000003) that does not have any new above-ground shoot-borne shoot systems (PPO:0001004).

new shoot system absent

OBO ID: PPO:0002600

IRI: http://purl.obolibrary.org/obo/PPO_0002600

Definition: A 'new shoot system presence' (PPO:0002003) trait that is a 'quality of' (RO:0000080) a 'whole plant' (PO:0000003) that does not have any new shoot systems (PPO:0001003).

non-dormant leaf buds absent

OBO ID: PPO:0002608

IRI: http://purl.obolibrary.org/obo/PPO_0002608

Definition: A 'non-dormant leaf bud presence' (PPO:0002011) trait that is a 'quality of' (RO:0000080) a 'whole plant' (PO:0000003) that does not have any non-dormant leaf buds (PPO:0001010).

leaf buds absent

OBO ID: PPO:0002606

IRI: http://purl.obolibrary.org/obo/PPO_0002606

Definition: A 'leaf bud presence' (PPO:0002009) trait that is a 'quality of' (RO:0000080) a 'whole plant' (PO:0000003) that does not have any vegetative buds (PO:0000058).

unfolding true leaves absent

OBO ID: PPO:0002613

IRI: http://purl.obolibrary.org/obo/PPO_0002613

Definition: An 'unfolding true leaf presence' (PPO:0002016) trait that is a 'quality of' (RO:0000080) a 'whole plant' (PO:0000003) that does not have any unfolding true leaves (PPO:0001014).

expanding true leaves absent

OBO ID: PPO:0002621

IRI: http://purl.obolibrary.org/obo/PPO_0002621

Definition: An 'expanding true leaf presence' (PPO:0002024) trait that is a 'quality of' (RO:0000080) a 'whole plant' (PO:0000003) that does not have any expanding true leaves (PPO:0001022).

true leaves absent

OBO ID: PPO:0002612

IRI: http://purl.obolibrary.org/obo/PPO_0002612

Definition: A 'true leaf presence' (PPO:0002015) trait that is a 'quality of' (RO:0000080) a 'whole plant' (PO:0000003) that does not have any true leaves (PPO:0001013).

vascular leaves absent

OBO ID: PPO:0002611

IRI: http://purl.obolibrary.org/obo/PPO_0002611

Definition: A 'vascular leaf presence' (PPO:0002014) trait that is a 'quality of' (RO:0000080) a 'whole plant' (PO:0000003) that does not have any vascular leaves (PO:0009025).

cones absent

OBO ID: PPO:0002645

IRI: http://purl.obolibrary.org/obo/PPO_0002645

Definition: A 'cone presence' (PPO:0002048) trait that is a 'quality of' (RO:0000080) a 'whole plant' (PO:0000003) that does not have any cones (PO:0025083).

reproductive structures absent

OBO ID: PPO:0002622

IRI: http://purl.obolibrary.org/obo/PPO_0002622

Definition: A 'reproductive structure presence' (PPO:0002025) trait that is a 'quality of' (RO:0000080) a 'whole plant' (PO:0000003) that does not have any reproductive structures (PPO:0001023).

dormant leaf buds absent

OBO ID: PPO:0002607

IRI: http://purl.obolibrary.org/obo/PPO_0002607

Definition: A 'dormant leaf bud presence' (PPO:0002010) trait that is a 'quality of' (RO:0000080) a 'whole plant' (PO:0000003) that does not have any dormant leaf buds (PPO:0001009).

leaf buds absent

OBO ID: PPO:0002606

IRI: http://purl.obolibrary.org/obo/PPO_0002606

Definition: A 'leaf bud presence' (PPO:0002009) trait that is a 'quality of' (RO:0000080) a 'whole plant' (PO:0000003) that does not have any vegetative buds (PO:0000058).

expanded immature true leaves absent

OBO ID: PPO:0002620

IRI: http://purl.obolibrary.org/obo/PPO_0002620

Definition: An 'expanded immature true leaf presence' (PPO:0002023) trait that is a 'quality of' (RO:0000080) a 'whole plant' (PO:0000003) that does not have any expanded immature true leaves (PPO:0001021).

immature unfolded true leaves absent

OBO ID: PPO:0002617

IRI: http://purl.obolibrary.org/obo/PPO_0002617

Definition: An 'immature unfolded true leaf presence' (PPO:0002020) trait that is a 'quality of' (RO:0000080) a 'whole plant' (PO:0000003) that does not have any immature unfolded true leaves (PPO:0001018).

non-senescing unfolded true leaves absent

OBO ID: PPO:0002615

IRI: http://purl.obolibrary.org/obo/PPO_0002615

Definition: A 'non-senescing unfolded true leaf presence' (PPO:0002018) trait that is a 'quality of' (RO:0000080) a 'whole plant' (PO:0000003) that does not have any non-senescing unfolded true leaves (PPO:0001016).

unfolded true leaves absent

OBO ID: PPO:0002614

IRI: http://purl.obolibrary.org/obo/PPO_0002614

Definition: An 'unfolded true leaf presence' (PPO:0002017) trait that is a 'quality of' (RO:0000080) a 'whole plant' (PO:0000003) that does not have any unfolded true leaves (PPO:0001015).

true leaves absent

OBO ID: PPO:0002612

IRI: http://purl.obolibrary.org/obo/PPO_0002612

Definition: A 'true leaf presence' (PPO:0002015) trait that is a 'quality of' (RO:0000080) a 'whole plant' (PO:0000003) that does not have any true leaves (PPO:0001013).

vascular leaves absent

OBO ID: PPO:0002611

IRI: http://purl.obolibrary.org/obo/PPO_0002611

Definition: A 'vascular leaf presence' (PPO:0002014) trait that is a 'quality of' (RO:0000080) a 'whole plant' (PO:0000003) that does not have any vascular leaves (PO:0009025).

expanding true leaves absent

OBO ID: PPO:0002621

IRI: http://purl.obolibrary.org/obo/PPO_0002621

Definition: An 'expanding true leaf presence' (PPO:0002024) trait that is a 'quality of' (RO:0000080) a 'whole plant' (PO:0000003) that does not have any expanding true leaves (PPO:0001022).

true leaves absent

OBO ID: PPO:0002612

IRI: http://purl.obolibrary.org/obo/PPO_0002612

Definition: A 'true leaf presence' (PPO:0002015) trait that is a 'quality of' (RO:0000080) a 'whole plant' (PO:0000003) that does not have any true leaves (PPO:0001013).

vascular leaves absent

OBO ID: PPO:0002611

IRI: http://purl.obolibrary.org/obo/PPO_0002611

Definition: A 'vascular leaf presence' (PPO:0002014) trait that is a 'quality of' (RO:0000080) a 'whole plant' (PO:0000003) that does not have any vascular leaves (PO:0009025).

expanding unfolded true leaves absent

OBO ID: PPO:0002619

IRI: http://purl.obolibrary.org/obo/PPO_0002619

Definition: An 'expanding unfolded true leaf presence' (PPO:0002022) trait that is a 'quality of' (RO:0000080) a 'whole plant' (PO:0000003) that does not have any expanding unfolded true leaves (PPO:0001020).

expanding true leaves absent

OBO ID: PPO:0002621

IRI: http://purl.obolibrary.org/obo/PPO_0002621

Definition: An 'expanding true leaf presence' (PPO:0002024) trait that is a 'quality of' (RO:0000080) a 'whole plant' (PO:0000003) that does not have any expanding true leaves (PPO:0001022).

true leaves absent

OBO ID: PPO:0002612

IRI: http://purl.obolibrary.org/obo/PPO_0002612

Definition: A 'true leaf presence' (PPO:0002015) trait that is a 'quality of' (RO:0000080) a 'whole plant' (PO:0000003) that does not have any true leaves (PPO:0001013).

vascular leaves absent

OBO ID: PPO:0002611

IRI: http://purl.obolibrary.org/obo/PPO_0002611

Definition: A 'vascular leaf presence' (PPO:0002014) trait that is a 'quality of' (RO:0000080) a 'whole plant' (PO:0000003) that does not have any vascular leaves (PO:0009025).

immature unfolded true leaves absent

OBO ID: PPO:0002617

IRI: http://purl.obolibrary.org/obo/PPO_0002617

Definition: An 'immature unfolded true leaf presence' (PPO:0002020) trait that is a 'quality of' (RO:0000080) a 'whole plant' (PO:0000003) that does not have any immature unfolded true leaves (PPO:0001018).

non-senescing unfolded true leaves absent

OBO ID: PPO:0002615

IRI: http://purl.obolibrary.org/obo/PPO_0002615

Definition: A 'non-senescing unfolded true leaf presence' (PPO:0002018) trait that is a 'quality of' (RO:0000080) a 'whole plant' (PO:0000003) that does not have any non-senescing unfolded true leaves (PPO:0001016).

unfolded true leaves absent

OBO ID: PPO:0002614

IRI: http://purl.obolibrary.org/obo/PPO_0002614

Definition: An 'unfolded true leaf presence' (PPO:0002017) trait that is a 'quality of' (RO:0000080) a 'whole plant' (PO:0000003) that does not have any unfolded true leaves (PPO:0001015).

true leaves absent

OBO ID: PPO:0002612

IRI: http://purl.obolibrary.org/obo/PPO_0002612

Definition: A 'true leaf presence' (PPO:0002015) trait that is a 'quality of' (RO:0000080) a 'whole plant' (PO:0000003) that does not have any true leaves (PPO:0001013).

vascular leaves absent

OBO ID: PPO:0002611

IRI: http://purl.obolibrary.org/obo/PPO_0002611

Definition: A 'vascular leaf presence' (PPO:0002014) trait that is a 'quality of' (RO:0000080) a 'whole plant' (PO:0000003) that does not have any vascular leaves (PO:0009025).

floral structures absent

OBO ID: PPO:0002623

IRI: http://purl.obolibrary.org/obo/PPO_0002623

Definition: A 'floral structure presence' (PPO:0002026) trait that is a 'quality of' (RO:0000080) a 'whole plant' (PO:0000003) that does not have any floral structures (PPO:0001025).

reproductive structures absent

OBO ID: PPO:0002622

IRI: http://purl.obolibrary.org/obo/PPO_0002622

Definition: A 'reproductive structure presence' (PPO:0002025) trait that is a 'quality of' (RO:0000080) a 'whole plant' (PO:0000003) that does not have any reproductive structures (PPO:0001023).

flower heads absent

OBO ID: PPO:0002635

IRI: http://purl.obolibrary.org/obo/PPO_0002635

Definition: A 'flower head presence' (PPO:0002038) trait that is a 'quality of' (RO:0000080) a 'whole plant' (PO:0000003) that does not have any PO:inflorescences (PO:0009049).

floral structures absent

OBO ID: PPO:0002623

IRI: http://purl.obolibrary.org/obo/PPO_0002623

Definition: A 'floral structure presence' (PPO:0002026) trait that is a 'quality of' (RO:0000080) a 'whole plant' (PO:0000003) that does not have any floral structures (PPO:0001025).

reproductive structures absent

OBO ID: PPO:0002622

IRI: http://purl.obolibrary.org/obo/PPO_0002622

Definition: A 'reproductive structure presence' (PPO:0002025) trait that is a 'quality of' (RO:0000080) a 'whole plant' (PO:0000003) that does not have any reproductive structures (PPO:0001023).

flowers absent

OBO ID: PPO:0002629

IRI: http://purl.obolibrary.org/obo/PPO_0002629

Definition: A 'flower presence' (PPO:0002032) trait that is a 'quality of' (RO:0000080) a 'whole plant' (PO:0000003) that does not have any PO:flowers (PO:0009046).

floral structures absent

OBO ID: PPO:0002623

IRI: http://purl.obolibrary.org/obo/PPO_0002623

Definition: A 'floral structure presence' (PPO:0002026) trait that is a 'quality of' (RO:0000080) a 'whole plant' (PO:0000003) that does not have any floral structures (PPO:0001025).

reproductive structures absent

OBO ID: PPO:0002622

IRI: http://purl.obolibrary.org/obo/PPO_0002622

Definition: A 'reproductive structure presence' (PPO:0002025) trait that is a 'quality of' (RO:0000080) a 'whole plant' (PO:0000003) that does not have any reproductive structures (PPO:0001023).

fresh pollen cones absent

OBO ID: PPO:0002647

IRI: http://purl.obolibrary.org/obo/PPO_0002647

Definition: A 'fresh pollen cone presence' (PPO:0002050) trait that is a 'quality of' (RO:0000080) a 'whole plant' (PO:0000003) that does not have any fresh pollen cones (PPO:0001046).

pollen cones absent

OBO ID: PPO:0002646

IRI: http://purl.obolibrary.org/obo/PPO_0002646

Definition: A 'pollen cone presence' (PPO:0002049) trait that is a 'quality of' (RO:0000080) a 'whole plant' (PO:0000003) that does not have any pollen cones (PO:0005031).

cones absent

OBO ID: PPO:0002645

IRI: http://purl.obolibrary.org/obo/PPO_0002645

Definition: A 'cone presence' (PPO:0002048) trait that is a 'quality of' (RO:0000080) a 'whole plant' (PO:0000003) that does not have any cones (PO:0025083).

reproductive structures absent

OBO ID: PPO:0002622

IRI: http://purl.obolibrary.org/obo/PPO_0002622

Definition: A 'reproductive structure presence' (PPO:0002025) trait that is a 'quality of' (RO:0000080) a 'whole plant' (PO:0000003) that does not have any reproductive structures (PPO:0001023).

fresh seed cones absent

OBO ID: PPO:0002651

IRI: http://purl.obolibrary.org/obo/PPO_0002651

Definition: A 'fresh seed cone presence' (PPO:0002054) trait that is a 'quality of' (RO:0000080) a 'whole plant' (PO:0000003) that does not have any fresh seed cones (PPO:0001049).

seed cones absent

OBO ID: PPO:0002650

IRI: http://purl.obolibrary.org/obo/PPO_0002650

Definition: A 'seed cone presence' (PPO:0002053) trait that is a 'quality of' (RO:0000080) a 'whole plant' (PO:0000003) that does not have any seed cones (PO:0005032).

cones absent

OBO ID: PPO:0002645

IRI: http://purl.obolibrary.org/obo/PPO_0002645

Definition: A 'cone presence' (PPO:0002048) trait that is a 'quality of' (RO:0000080) a 'whole plant' (PO:0000003) that does not have any cones (PO:0025083).

reproductive structures absent

OBO ID: PPO:0002622

IRI: http://purl.obolibrary.org/obo/PPO_0002622

Definition: A 'reproductive structure presence' (PPO:0002025) trait that is a 'quality of' (RO:0000080) a 'whole plant' (PO:0000003) that does not have any reproductive structures (PPO:0001023).

fruits absent

OBO ID: PPO:0002641

IRI: http://purl.obolibrary.org/obo/PPO_0002641

Definition: A 'fruit presence' (PPO:0002044) trait that is a 'quality of' (RO:0000080) a 'whole plant' (PO:0000003) that does not have any simple fruits or compound fruits (PPO:0001042).

reproductive structures absent

OBO ID: PPO:0002622

IRI: http://purl.obolibrary.org/obo/PPO_0002622

Definition: A 'reproductive structure presence' (PPO:0002025) trait that is a 'quality of' (RO:0000080) a 'whole plant' (PO:0000003) that does not have any reproductive structures (PPO:0001023).

immature unfolded true leaves absent

OBO ID: PPO:0002617

IRI: http://purl.obolibrary.org/obo/PPO_0002617

Definition: An 'immature unfolded true leaf presence' (PPO:0002020) trait that is a 'quality of' (RO:0000080) a 'whole plant' (PO:0000003) that does not have any immature unfolded true leaves (PPO:0001018).

non-senescing unfolded true leaves absent

OBO ID: PPO:0002615

IRI: http://purl.obolibrary.org/obo/PPO_0002615

Definition: A 'non-senescing unfolded true leaf presence' (PPO:0002018) trait that is a 'quality of' (RO:0000080) a 'whole plant' (PO:0000003) that does not have any non-senescing unfolded true leaves (PPO:0001016).

unfolded true leaves absent

OBO ID: PPO:0002614

IRI: http://purl.obolibrary.org/obo/PPO_0002614

Definition: An 'unfolded true leaf presence' (PPO:0002017) trait that is a 'quality of' (RO:0000080) a 'whole plant' (PO:0000003) that does not have any unfolded true leaves (PPO:0001015).

true leaves absent

OBO ID: PPO:0002612

IRI: http://purl.obolibrary.org/obo/PPO_0002612

Definition: A 'true leaf presence' (PPO:0002015) trait that is a 'quality of' (RO:0000080) a 'whole plant' (PO:0000003) that does not have any true leaves (PPO:0001013).

vascular leaves absent

OBO ID: PPO:0002611

IRI: http://purl.obolibrary.org/obo/PPO_0002611

Definition: A 'vascular leaf presence' (PPO:0002014) trait that is a 'quality of' (RO:0000080) a 'whole plant' (PO:0000003) that does not have any vascular leaves (PO:0009025).

leaf buds absent

OBO ID: PPO:0002606

IRI: http://purl.obolibrary.org/obo/PPO_0002606

Definition: A 'leaf bud presence' (PPO:0002009) trait that is a 'quality of' (RO:0000080) a 'whole plant' (PO:0000003) that does not have any vegetative buds (PO:0000058).

mature true leaves absent

OBO ID: PPO:0002618

IRI: http://purl.obolibrary.org/obo/PPO_0002618

Definition: A 'mature true leaf presence' (PPO:0002021) trait that is a 'quality of' (RO:0000080) a 'whole plant' (PO:0000003) that does not have any mature true leaves (PPO:0001019).

non-senescing unfolded true leaves absent

OBO ID: PPO:0002615

IRI: http://purl.obolibrary.org/obo/PPO_0002615

Definition: A 'non-senescing unfolded true leaf presence' (PPO:0002018) trait that is a 'quality of' (RO:0000080) a 'whole plant' (PO:0000003) that does not have any non-senescing unfolded true leaves (PPO:0001016).

unfolded true leaves absent

OBO ID: PPO:0002614

IRI: http://purl.obolibrary.org/obo/PPO_0002614

Definition: An 'unfolded true leaf presence' (PPO:0002017) trait that is a 'quality of' (RO:0000080) a 'whole plant' (PO:0000003) that does not have any unfolded true leaves (PPO:0001015).

true leaves absent

OBO ID: PPO:0002612

IRI: http://purl.obolibrary.org/obo/PPO_0002612

Definition: A 'true leaf presence' (PPO:0002015) trait that is a 'quality of' (RO:0000080) a 'whole plant' (PO:0000003) that does not have any true leaves (PPO:0001013).

vascular leaves absent

OBO ID: PPO:0002611

IRI: http://purl.obolibrary.org/obo/PPO_0002611

Definition: A 'vascular leaf presence' (PPO:0002014) trait that is a 'quality of' (RO:0000080) a 'whole plant' (PO:0000003) that does not have any vascular leaves (PO:0009025).

new above-ground shoot-borne shoot systems absent

OBO ID: PPO:0002601

IRI: http://purl.obolibrary.org/obo/PPO_0002601

Definition: A 'new above-ground shoot-borne shoot system presence' (PPO:0002004) trait that is a 'quality of' (RO:0000080) a 'whole plant' (PO:0000003) that does not have any new above-ground shoot-borne shoot systems (PPO:0001004).

new shoot system absent

OBO ID: PPO:0002600

IRI: http://purl.obolibrary.org/obo/PPO_0002600

Definition: A 'new shoot system presence' (PPO:0002003) trait that is a 'quality of' (RO:0000080) a 'whole plant' (PO:0000003) that does not have any new shoot systems (PPO:0001003).

new shoot system absent

OBO ID: PPO:0002600

IRI: http://purl.obolibrary.org/obo/PPO_0002600

Definition: A 'new shoot system presence' (PPO:0002003) trait that is a 'quality of' (RO:0000080) a 'whole plant' (PO:0000003) that does not have any new shoot systems (PPO:0001003).

new shoot systems emerging from ground absent

OBO ID: PPO:0002602

IRI: http://purl.obolibrary.org/obo/PPO_0002602

Definition: A 'new shoot system emerging from ground presence' (PPO:0002005) trait that is a 'quality of' (RO:0000080) a 'whole plant' (PO:0000003) that does not have any new shoot systems emerging from ground (PPO:0001005).

new shoot system absent

OBO ID: PPO:0002600

IRI: http://purl.obolibrary.org/obo/PPO_0002600

Definition: A 'new shoot system presence' (PPO:0002003) trait that is a 'quality of' (RO:0000080) a 'whole plant' (PO:0000003) that does not have any new shoot systems (PPO:0001003).

new shoot systems emerging from ground in first growth cycle absent

OBO ID: PPO:0002603

IRI: http://purl.obolibrary.org/obo/PPO_0002603

Definition: A 'new shoot system emerging from ground in first growth cycle presence' (PPO:0002006) trait that is a 'quality of' (RO:0000080) a 'whole plant' (PO:0000003) that does not have any new shoot systems emerging from ground in first growth cycle (PPO:0001006).

new shoot systems emerging from ground absent

OBO ID: PPO:0002602

IRI: http://purl.obolibrary.org/obo/PPO_0002602

Definition: A 'new shoot system emerging from ground presence' (PPO:0002005) trait that is a 'quality of' (RO:0000080) a 'whole plant' (PO:0000003) that does not have any new shoot systems emerging from ground (PPO:0001005).

new shoot system absent

OBO ID: PPO:0002600

IRI: http://purl.obolibrary.org/obo/PPO_0002600

Definition: A 'new shoot system presence' (PPO:0002003) trait that is a 'quality of' (RO:0000080) a 'whole plant' (PO:0000003) that does not have any new shoot systems (PPO:0001003).

new shoot systems emerging from ground in later growth cycle absent

OBO ID: PPO:0002605

IRI: http://purl.obolibrary.org/obo/PPO_0002605

Definition: A 'new shoot system emerging from ground in later growth cycle presence' (PPO:0002008) trait that is a 'quality of' (RO:0000080) a 'whole plant' (PO:0000003) that does not have any new shoot systems emerging from ground in later growth cycle (PPO:0001008).

new shoot systems emerging from ground absent

OBO ID: PPO:0002602

IRI: http://purl.obolibrary.org/obo/PPO_0002602

Definition: A 'new shoot system emerging from ground presence' (PPO:0002005) trait that is a 'quality of' (RO:0000080) a 'whole plant' (PO:0000003) that does not have any new shoot systems emerging from ground (PPO:0001005).

new shoot system absent

OBO ID: PPO:0002600

IRI: http://purl.obolibrary.org/obo/PPO_0002600

Definition: A 'new shoot system presence' (PPO:0002003) trait that is a 'quality of' (RO:0000080) a 'whole plant' (PO:0000003) that does not have any new shoot systems (PPO:0001003).

non-dormant leaf buds absent

OBO ID: PPO:0002608

IRI: http://purl.obolibrary.org/obo/PPO_0002608

Definition: A 'non-dormant leaf bud presence' (PPO:0002011) trait that is a 'quality of' (RO:0000080) a 'whole plant' (PO:0000003) that does not have any non-dormant leaf buds (PPO:0001010).

leaf buds absent

OBO ID: PPO:0002606

IRI: http://purl.obolibrary.org/obo/PPO_0002606

Definition: A 'leaf bud presence' (PPO:0002009) trait that is a 'quality of' (RO:0000080) a 'whole plant' (PO:0000003) that does not have any vegetative buds (PO:0000058).

non-senesced floral structures absent

OBO ID: PPO:0002624

IRI: http://purl.obolibrary.org/obo/PPO_0002624

Definition: A 'non-senesced floral structure presence' (PPO:0002027) trait that is a 'quality of' (RO:0000080) a 'whole plant' (PO:0000003) that does not have any non-senesced floral structures (PPO:0001026).

floral structures absent

OBO ID: PPO:0002623

IRI: http://purl.obolibrary.org/obo/PPO_0002623

Definition: A 'floral structure presence' (PPO:0002026) trait that is a 'quality of' (RO:0000080) a 'whole plant' (PO:0000003) that does not have any floral structures (PPO:0001025).

reproductive structures absent

OBO ID: PPO:0002622

IRI: http://purl.obolibrary.org/obo/PPO_0002622

Definition: A 'reproductive structure presence' (PPO:0002025) trait that is a 'quality of' (RO:0000080) a 'whole plant' (PO:0000003) that does not have any reproductive structures (PPO:0001023).

non-senesced flower heads absent

OBO ID: PPO:0002636

IRI: http://purl.obolibrary.org/obo/PPO_0002636

Definition: A 'non-senesced flower head presence' (PPO:0002039) trait that is a 'quality of' (RO:0000080) a 'whole plant' (PO:0000003) that does not have any non-senesced flower heads (PPO:0001036).

flower heads absent

OBO ID: PPO:0002635

IRI: http://purl.obolibrary.org/obo/PPO_0002635

Definition: A 'flower head presence' (PPO:0002038) trait that is a 'quality of' (RO:0000080) a 'whole plant' (PO:0000003) that does not have any PO:inflorescences (PO:0009049).

floral structures absent

OBO ID: PPO:0002623

IRI: http://purl.obolibrary.org/obo/PPO_0002623

Definition: A 'floral structure presence' (PPO:0002026) trait that is a 'quality of' (RO:0000080) a 'whole plant' (PO:0000003) that does not have any floral structures (PPO:0001025).

reproductive structures absent

OBO ID: PPO:0002622

IRI: http://purl.obolibrary.org/obo/PPO_0002622

Definition: A 'reproductive structure presence' (PPO:0002025) trait that is a 'quality of' (RO:0000080) a 'whole plant' (PO:0000003) that does not have any reproductive structures (PPO:0001023).

non-senesced flowers absent

OBO ID: PPO:0002630

IRI: http://purl.obolibrary.org/obo/PPO_0002630

Definition: A 'non-senesced flower presence' (PPO:0002033) trait that is a 'quality of' (RO:0000080) a 'whole plant' (PO:0000003) that does not have any non-senesced flowers (PPO:0001031).

flowers absent

OBO ID: PPO:0002629

IRI: http://purl.obolibrary.org/obo/PPO_0002629

Definition: A 'flower presence' (PPO:0002032) trait that is a 'quality of' (RO:0000080) a 'whole plant' (PO:0000003) that does not have any PO:flowers (PO:0009046).

floral structures absent

OBO ID: PPO:0002623

IRI: http://purl.obolibrary.org/obo/PPO_0002623

Definition: A 'floral structure presence' (PPO:0002026) trait that is a 'quality of' (RO:0000080) a 'whole plant' (PO:0000003) that does not have any floral structures (PPO:0001025).

reproductive structures absent

OBO ID: PPO:0002622

IRI: http://purl.obolibrary.org/obo/PPO_0002622

Definition: A 'reproductive structure presence' (PPO:0002025) trait that is a 'quality of' (RO:0000080) a 'whole plant' (PO:0000003) that does not have any reproductive structures (PPO:0001023).

non-senescing unfolded true leaves absent

OBO ID: PPO:0002615

IRI: http://purl.obolibrary.org/obo/PPO_0002615

Definition: A 'non-senescing unfolded true leaf presence' (PPO:0002018) trait that is a 'quality of' (RO:0000080) a 'whole plant' (PO:0000003) that does not have any non-senescing unfolded true leaves (PPO:0001016).

unfolded true leaves absent

OBO ID: PPO:0002614

IRI: http://purl.obolibrary.org/obo/PPO_0002614

Definition: An 'unfolded true leaf presence' (PPO:0002017) trait that is a 'quality of' (RO:0000080) a 'whole plant' (PO:0000003) that does not have any unfolded true leaves (PPO:0001015).

true leaves absent

OBO ID: PPO:0002612

IRI: http://purl.obolibrary.org/obo/PPO_0002612

Definition: A 'true leaf presence' (PPO:0002015) trait that is a 'quality of' (RO:0000080) a 'whole plant' (PO:0000003) that does not have any true leaves (PPO:0001013).

vascular leaves absent

OBO ID: PPO:0002611

IRI: http://purl.obolibrary.org/obo/PPO_0002611

Definition: A 'vascular leaf presence' (PPO:0002014) trait that is a 'quality of' (RO:0000080) a 'whole plant' (PO:0000003) that does not have any vascular leaves (PO:0009025).

open floral structures absent

OBO ID: PPO:0002626

IRI: http://purl.obolibrary.org/obo/PPO_0002626

Definition: An 'open floral structure presence' (PPO:0002029) trait that is a 'quality of' (RO:0000080) a 'whole plant' (PO:0000003) that does not have any open floral structures (PPO:0001028).

flowers absent

OBO ID: PPO:0002629

IRI: http://purl.obolibrary.org/obo/PPO_0002629

Definition: A 'flower presence' (PPO:0002032) trait that is a 'quality of' (RO:0000080) a 'whole plant' (PO:0000003) that does not have any PO:flowers (PO:0009046).

floral structures absent

OBO ID: PPO:0002623

IRI: http://purl.obolibrary.org/obo/PPO_0002623

Definition: A 'floral structure presence' (PPO:0002026) trait that is a 'quality of' (RO:0000080) a 'whole plant' (PO:0000003) that does not have any floral structures (PPO:0001025).

reproductive structures absent

OBO ID: PPO:0002622

IRI: http://purl.obolibrary.org/obo/PPO_0002622

Definition: A 'reproductive structure presence' (PPO:0002025) trait that is a 'quality of' (RO:0000080) a 'whole plant' (PO:0000003) that does not have any reproductive structures (PPO:0001023).

non-senesced floral structures absent

OBO ID: PPO:0002624

IRI: http://purl.obolibrary.org/obo/PPO_0002624

Definition: A 'non-senesced floral structure presence' (PPO:0002027) trait that is a 'quality of' (RO:0000080) a 'whole plant' (PO:0000003) that does not have any non-senesced floral structures (PPO:0001026).

floral structures absent

OBO ID: PPO:0002623

IRI: http://purl.obolibrary.org/obo/PPO_0002623

Definition: A 'floral structure presence' (PPO:0002026) trait that is a 'quality of' (RO:0000080) a 'whole plant' (PO:0000003) that does not have any floral structures (PPO:0001025).

reproductive structures absent

OBO ID: PPO:0002622

IRI: http://purl.obolibrary.org/obo/PPO_0002622

Definition: A 'reproductive structure presence' (PPO:0002025) trait that is a 'quality of' (RO:0000080) a 'whole plant' (PO:0000003) that does not have any reproductive structures (PPO:0001023).

open flower heads absent

OBO ID: PPO:0002638

IRI: http://purl.obolibrary.org/obo/PPO_0002638

Definition: An 'open flower head presence' (PPO:0002041) trait that is a 'quality of' (RO:0000080) a 'whole plant' (PO:0000003) that does not have any open flower heads (PPO:0001038).

non-senesced flower heads absent

OBO ID: PPO:0002636

IRI: http://purl.obolibrary.org/obo/PPO_0002636

Definition: A 'non-senesced flower head presence' (PPO:0002039) trait that is a 'quality of' (RO:0000080) a 'whole plant' (PO:0000003) that does not have any non-senesced flower heads (PPO:0001036).

flower heads absent

OBO ID: PPO:0002635

IRI: http://purl.obolibrary.org/obo/PPO_0002635

Definition: A 'flower head presence' (PPO:0002038) trait that is a 'quality of' (RO:0000080) a 'whole plant' (PO:0000003) that does not have any PO:inflorescences (PO:0009049).

floral structures absent

OBO ID: PPO:0002623

IRI: http://purl.obolibrary.org/obo/PPO_0002623

Definition: A 'floral structure presence' (PPO:0002026) trait that is a 'quality of' (RO:0000080) a 'whole plant' (PO:0000003) that does not have any floral structures (PPO:0001025).

reproductive structures absent

OBO ID: PPO:0002622

IRI: http://purl.obolibrary.org/obo/PPO_0002622

Definition: A 'reproductive structure presence' (PPO:0002025) trait that is a 'quality of' (RO:0000080) a 'whole plant' (PO:0000003) that does not have any reproductive structures (PPO:0001023).

open flowers absent

OBO ID: PPO:0002632

IRI: http://purl.obolibrary.org/obo/PPO_0002632

Definition: An 'open flower presence' (PPO:0002035) trait that is a 'quality of' (RO:0000080) a 'whole plant' (PO:0000003) that does not have any open flowers (PPO:0001033).

non-senesced flowers absent

OBO ID: PPO:0002630

IRI: http://purl.obolibrary.org/obo/PPO_0002630

Definition: A 'non-senesced flower presence' (PPO:0002033) trait that is a 'quality of' (RO:0000080) a 'whole plant' (PO:0000003) that does not have any non-senesced flowers (PPO:0001031).

flowers absent

OBO ID: PPO:0002629

IRI: http://purl.obolibrary.org/obo/PPO_0002629

Definition: A 'flower presence' (PPO:0002032) trait that is a 'quality of' (RO:0000080) a 'whole plant' (PO:0000003) that does not have any PO:flowers (PO:0009046).

floral structures absent

OBO ID: PPO:0002623

IRI: http://purl.obolibrary.org/obo/PPO_0002623

Definition: A 'floral structure presence' (PPO:0002026) trait that is a 'quality of' (RO:0000080) a 'whole plant' (PO:0000003) that does not have any floral structures (PPO:0001025).

reproductive structures absent

OBO ID: PPO:0002622

IRI: http://purl.obolibrary.org/obo/PPO_0002622

Definition: A 'reproductive structure presence' (PPO:0002025) trait that is a 'quality of' (RO:0000080) a 'whole plant' (PO:0000003) that does not have any reproductive structures (PPO:0001023).

open flowers absent

OBO ID: PPO:0002632

IRI: http://purl.obolibrary.org/obo/PPO_0002632

Definition: An 'open flower presence' (PPO:0002035) trait that is a 'quality of' (RO:0000080) a 'whole plant' (PO:0000003) that does not have any open flowers (PPO:0001033).

non-senesced flowers absent

OBO ID: PPO:0002630

IRI: http://purl.obolibrary.org/obo/PPO_0002630

Definition: A 'non-senesced flower presence' (PPO:0002033) trait that is a 'quality of' (RO:0000080) a 'whole plant' (PO:0000003) that does not have any non-senesced flowers (PPO:0001031).

flowers absent

OBO ID: PPO:0002629

IRI: http://purl.obolibrary.org/obo/PPO_0002629

Definition: A 'flower presence' (PPO:0002032) trait that is a 'quality of' (RO:0000080) a 'whole plant' (PO:0000003) that does not have any PO:flowers (PO:0009046).

floral structures absent

OBO ID: PPO:0002623

IRI: http://purl.obolibrary.org/obo/PPO_0002623

Definition: A 'floral structure presence' (PPO:0002026) trait that is a 'quality of' (RO:0000080) a 'whole plant' (PO:0000003) that does not have any floral structures (PPO:0001025).

reproductive structures absent

OBO ID: PPO:0002622

IRI: http://purl.obolibrary.org/obo/PPO_0002622

Definition: A 'reproductive structure presence' (PPO:0002025) trait that is a 'quality of' (RO:0000080) a 'whole plant' (PO:0000003) that does not have any reproductive structures (PPO:0001023).

open pollen cones absent

OBO ID: PPO:0002648

IRI: http://purl.obolibrary.org/obo/PPO_0002648

Definition: An 'open pollen cone presence' (PPO:0002051) trait that is a 'quality of' (RO:0000080) a 'whole plant' (PO:0000003) that does not have any open pollen cones (PPO:0001047).

fresh pollen cones absent

OBO ID: PPO:0002647

IRI: http://purl.obolibrary.org/obo/PPO_0002647

Definition: A 'fresh pollen cone presence' (PPO:0002050) trait that is a 'quality of' (RO:0000080) a 'whole plant' (PO:0000003) that does not have any fresh pollen cones (PPO:0001046).

pollen cones absent

OBO ID: PPO:0002646

IRI: http://purl.obolibrary.org/obo/PPO_0002646

Definition: A 'pollen cone presence' (PPO:0002049) trait that is a 'quality of' (RO:0000080) a 'whole plant' (PO:0000003) that does not have any pollen cones (PO:0005031).

cones absent

OBO ID: PPO:0002645

IRI: http://purl.obolibrary.org/obo/PPO_0002645

Definition: A 'cone presence' (PPO:0002048) trait that is a 'quality of' (RO:0000080) a 'whole plant' (PO:0000003) that does not have any cones (PO:0025083).

reproductive structures absent

OBO ID: PPO:0002622

IRI: http://purl.obolibrary.org/obo/PPO_0002622

Definition: A 'reproductive structure presence' (PPO:0002025) trait that is a 'quality of' (RO:0000080) a 'whole plant' (PO:0000003) that does not have any reproductive structures (PPO:0001023).

pollen cones absent

OBO ID: PPO:0002646

IRI: http://purl.obolibrary.org/obo/PPO_0002646

Definition: A 'pollen cone presence' (PPO:0002049) trait that is a 'quality of' (RO:0000080) a 'whole plant' (PO:0000003) that does not have any pollen cones (PO:0005031).

cones absent

OBO ID: PPO:0002645

IRI: http://purl.obolibrary.org/obo/PPO_0002645

Definition: A 'cone presence' (PPO:0002048) trait that is a 'quality of' (RO:0000080) a 'whole plant' (PO:0000003) that does not have any cones (PO:0025083).

reproductive structures absent

OBO ID: PPO:0002622

IRI: http://purl.obolibrary.org/obo/PPO_0002622

Definition: A 'reproductive structure presence' (PPO:0002025) trait that is a 'quality of' (RO:0000080) a 'whole plant' (PO:0000003) that does not have any reproductive structures (PPO:0001023).

pollen-releasing floral structures absent

OBO ID: PPO:0002627

IRI: http://purl.obolibrary.org/obo/PPO_0002627

Definition: A 'pollen-releasing floral structure presence' (PPO:0002030) trait that is a 'quality of' (RO:0000080) a 'whole plant' (PO:0000003) that does not have any pollen-releasing floral structures (PPO:0001029).

open floral structures absent

OBO ID: PPO:0002626

IRI: http://purl.obolibrary.org/obo/PPO_0002626

Definition: An 'open floral structure presence' (PPO:0002029) trait that is a 'quality of' (RO:0000080) a 'whole plant' (PO:0000003) that does not have any open floral structures (PPO:0001028).

flowers absent

OBO ID: PPO:0002629

IRI: http://purl.obolibrary.org/obo/PPO_0002629

Definition: A 'flower presence' (PPO:0002032) trait that is a 'quality of' (RO:0000080) a 'whole plant' (PO:0000003) that does not have any PO:flowers (PO:0009046).

floral structures absent

OBO ID: PPO:0002623

IRI: http://purl.obolibrary.org/obo/PPO_0002623

Definition: A 'floral structure presence' (PPO:0002026) trait that is a 'quality of' (RO:0000080) a 'whole plant' (PO:0000003) that does not have any floral structures (PPO:0001025).

reproductive structures absent

OBO ID: PPO:0002622

IRI: http://purl.obolibrary.org/obo/PPO_0002622

Definition: A 'reproductive structure presence' (PPO:0002025) trait that is a 'quality of' (RO:0000080) a 'whole plant' (PO:0000003) that does not have any reproductive structures (PPO:0001023).

non-senesced floral structures absent

OBO ID: PPO:0002624

IRI: http://purl.obolibrary.org/obo/PPO_0002624

Definition: A 'non-senesced floral structure presence' (PPO:0002027) trait that is a 'quality of' (RO:0000080) a 'whole plant' (PO:0000003) that does not have any non-senesced floral structures (PPO:0001026).

floral structures absent

OBO ID: PPO:0002623

IRI: http://purl.obolibrary.org/obo/PPO_0002623

Definition: A 'floral structure presence' (PPO:0002026) trait that is a 'quality of' (RO:0000080) a 'whole plant' (PO:0000003) that does not have any floral structures (PPO:0001025).

reproductive structures absent

OBO ID: PPO:0002622

IRI: http://purl.obolibrary.org/obo/PPO_0002622

Definition: A 'reproductive structure presence' (PPO:0002025) trait that is a 'quality of' (RO:0000080) a 'whole plant' (PO:0000003) that does not have any reproductive structures (PPO:0001023).

pollen-releasing flowers absent

OBO ID: PPO:0002633

IRI: http://purl.obolibrary.org/obo/PPO_0002633

Definition: A 'pollen-releasing flower presence' (PPO:0002036) trait that is a 'quality of' (RO:0000080) a 'whole plant' (PO:0000003) that does not have any pollen-releasing flowers (PPO:0001034).

open flowers absent

OBO ID: PPO:0002632

IRI: http://purl.obolibrary.org/obo/PPO_0002632

Definition: An 'open flower presence' (PPO:0002035) trait that is a 'quality of' (RO:0000080) a 'whole plant' (PO:0000003) that does not have any open flowers (PPO:0001033).

non-senesced flowers absent

OBO ID: PPO:0002630

IRI: http://purl.obolibrary.org/obo/PPO_0002630

Definition: A 'non-senesced flower presence' (PPO:0002033) trait that is a 'quality of' (RO:0000080) a 'whole plant' (PO:0000003) that does not have any non-senesced flowers (PPO:0001031).

flowers absent

OBO ID: PPO:0002629

IRI: http://purl.obolibrary.org/obo/PPO_0002629

Definition: A 'flower presence' (PPO:0002032) trait that is a 'quality of' (RO:0000080) a 'whole plant' (PO:0000003) that does not have any PO:flowers (PO:0009046).

floral structures absent

OBO ID: PPO:0002623

IRI: http://purl.obolibrary.org/obo/PPO_0002623

Definition: A 'floral structure presence' (PPO:0002026) trait that is a 'quality of' (RO:0000080) a 'whole plant' (PO:0000003) that does not have any floral structures (PPO:0001025).

reproductive structures absent

OBO ID: PPO:0002622

IRI: http://purl.obolibrary.org/obo/PPO_0002622

Definition: A 'reproductive structure presence' (PPO:0002025) trait that is a 'quality of' (RO:0000080) a 'whole plant' (PO:0000003) that does not have any reproductive structures (PPO:0001023).

pollen-releasing flower heads absent

OBO ID: PPO:0002639

IRI: http://purl.obolibrary.org/obo/PPO_0002639

Definition: A 'pollen-releasing flower head presence' (PPO:0002042) trait that is a 'quality of' (RO:0000080) a 'whole plant' (PO:0000003) that does not have any pollen-releasing flower heads (PPO:0001039).

open flower heads absent

OBO ID: PPO:0002638

IRI: http://purl.obolibrary.org/obo/PPO_0002638

Definition: An 'open flower head presence' (PPO:0002041) trait that is a 'quality of' (RO:0000080) a 'whole plant' (PO:0000003) that does not have any open flower heads (PPO:0001038).

non-senesced flower heads absent

OBO ID: PPO:0002636

IRI: http://purl.obolibrary.org/obo/PPO_0002636

Definition: A 'non-senesced flower head presence' (PPO:0002039) trait that is a 'quality of' (RO:0000080) a 'whole plant' (PO:0000003) that does not have any non-senesced flower heads (PPO:0001036).

flower heads absent

OBO ID: PPO:0002635

IRI: http://purl.obolibrary.org/obo/PPO_0002635

Definition: A 'flower head presence' (PPO:0002038) trait that is a 'quality of' (RO:0000080) a 'whole plant' (PO:0000003) that does not have any PO:inflorescences (PO:0009049).

floral structures absent

OBO ID: PPO:0002623

IRI: http://purl.obolibrary.org/obo/PPO_0002623

Definition: A 'floral structure presence' (PPO:0002026) trait that is a 'quality of' (RO:0000080) a 'whole plant' (PO:0000003) that does not have any floral structures (PPO:0001025).

reproductive structures absent

OBO ID: PPO:0002622

IRI: http://purl.obolibrary.org/obo/PPO_0002622

Definition: A 'reproductive structure presence' (PPO:0002025) trait that is a 'quality of' (RO:0000080) a 'whole plant' (PO:0000003) that does not have any reproductive structures (PPO:0001023).

open flowers absent

OBO ID: PPO:0002632

IRI: http://purl.obolibrary.org/obo/PPO_0002632

Definition: An 'open flower presence' (PPO:0002035) trait that is a 'quality of' (RO:0000080) a 'whole plant' (PO:0000003) that does not have any open flowers (PPO:0001033).

non-senesced flowers absent

OBO ID: PPO:0002630

IRI: http://purl.obolibrary.org/obo/PPO_0002630

Definition: A 'non-senesced flower presence' (PPO:0002033) trait that is a 'quality of' (RO:0000080) a 'whole plant' (PO:0000003) that does not have any non-senesced flowers (PPO:0001031).

flowers absent

OBO ID: PPO:0002629

IRI: http://purl.obolibrary.org/obo/PPO_0002629

Definition: A 'flower presence' (PPO:0002032) trait that is a 'quality of' (RO:0000080) a 'whole plant' (PO:0000003) that does not have any PO:flowers (PO:0009046).

floral structures absent

OBO ID: PPO:0002623

IRI: http://purl.obolibrary.org/obo/PPO_0002623

Definition: A 'floral structure presence' (PPO:0002026) trait that is a 'quality of' (RO:0000080) a 'whole plant' (PO:0000003) that does not have any floral structures (PPO:0001025).

reproductive structures absent

OBO ID: PPO:0002622

IRI: http://purl.obolibrary.org/obo/PPO_0002622

Definition: A 'reproductive structure presence' (PPO:0002025) trait that is a 'quality of' (RO:0000080) a 'whole plant' (PO:0000003) that does not have any reproductive structures (PPO:0001023).

pollen-releasing flowers absent

OBO ID: PPO:0002633

IRI: http://purl.obolibrary.org/obo/PPO_0002633

Definition: A 'pollen-releasing flower presence' (PPO:0002036) trait that is a 'quality of' (RO:0000080) a 'whole plant' (PO:0000003) that does not have any pollen-releasing flowers (PPO:0001034).

open flowers absent

OBO ID: PPO:0002632

IRI: http://purl.obolibrary.org/obo/PPO_0002632

Definition: An 'open flower presence' (PPO:0002035) trait that is a 'quality of' (RO:0000080) a 'whole plant' (PO:0000003) that does not have any open flowers (PPO:0001033).

non-senesced flowers absent

OBO ID: PPO:0002630

IRI: http://purl.obolibrary.org/obo/PPO_0002630

Definition: A 'non-senesced flower presence' (PPO:0002033) trait that is a 'quality of' (RO:0000080) a 'whole plant' (PO:0000003) that does not have any non-senesced flowers (PPO:0001031).

flowers absent

OBO ID: PPO:0002629

IRI: http://purl.obolibrary.org/obo/PPO_0002629

Definition: A 'flower presence' (PPO:0002032) trait that is a 'quality of' (RO:0000080) a 'whole plant' (PO:0000003) that does not have any PO:flowers (PO:0009046).

floral structures absent

OBO ID: PPO:0002623

IRI: http://purl.obolibrary.org/obo/PPO_0002623

Definition: A 'floral structure presence' (PPO:0002026) trait that is a 'quality of' (RO:0000080) a 'whole plant' (PO:0000003) that does not have any floral structures (PPO:0001025).

reproductive structures absent

OBO ID: PPO:0002622

IRI: http://purl.obolibrary.org/obo/PPO_0002622

Definition: A 'reproductive structure presence' (PPO:0002025) trait that is a 'quality of' (RO:0000080) a 'whole plant' (PO:0000003) that does not have any reproductive structures (PPO:0001023).

pollen-releasing flowers absent

OBO ID: PPO:0002633

IRI: http://purl.obolibrary.org/obo/PPO_0002633

Definition: A 'pollen-releasing flower presence' (PPO:0002036) trait that is a 'quality of' (RO:0000080) a 'whole plant' (PO:0000003) that does not have any pollen-releasing flowers (PPO:0001034).

open flowers absent

OBO ID: PPO:0002632

IRI: http://purl.obolibrary.org/obo/PPO_0002632

Definition: An 'open flower presence' (PPO:0002035) trait that is a 'quality of' (RO:0000080) a 'whole plant' (PO:0000003) that does not have any open flowers (PPO:0001033).

non-senesced flowers absent

OBO ID: PPO:0002630

IRI: http://purl.obolibrary.org/obo/PPO_0002630

Definition: A 'non-senesced flower presence' (PPO:0002033) trait that is a 'quality of' (RO:0000080) a 'whole plant' (PO:0000003) that does not have any non-senesced flowers (PPO:0001031).

flowers absent

OBO ID: PPO:0002629

IRI: http://purl.obolibrary.org/obo/PPO_0002629

Definition: A 'flower presence' (PPO:0002032) trait that is a 'quality of' (RO:0000080) a 'whole plant' (PO:0000003) that does not have any PO:flowers (PO:0009046).

floral structures absent

OBO ID: PPO:0002623

IRI: http://purl.obolibrary.org/obo/PPO_0002623

Definition: A 'floral structure presence' (PPO:0002026) trait that is a 'quality of' (RO:0000080) a 'whole plant' (PO:0000003) that does not have any floral structures (PPO:0001025).

reproductive structures absent

OBO ID: PPO:0002622

IRI: http://purl.obolibrary.org/obo/PPO_0002622

Definition: A 'reproductive structure presence' (PPO:0002025) trait that is a 'quality of' (RO:0000080) a 'whole plant' (PO:0000003) that does not have any reproductive structures (PPO:0001023).

pollen-releasing pollen cones absent

OBO ID: PPO:0002649

IRI: http://purl.obolibrary.org/obo/PPO_0002649

Definition: A 'pollen-releasing pollen cone presence' (PPO:0002052) trait that is a 'quality of' (RO:0000080) a 'whole plant' (PO:0000003) that does not have any pollen-releasing pollen cones (PPO:0001048).

open pollen cones absent

OBO ID: PPO:0002648

IRI: http://purl.obolibrary.org/obo/PPO_0002648

Definition: An 'open pollen cone presence' (PPO:0002051) trait that is a 'quality of' (RO:0000080) a 'whole plant' (PO:0000003) that does not have any open pollen cones (PPO:0001047).

fresh pollen cones absent

OBO ID: PPO:0002647

IRI: http://purl.obolibrary.org/obo/PPO_0002647

Definition: A 'fresh pollen cone presence' (PPO:0002050) trait that is a 'quality of' (RO:0000080) a 'whole plant' (PO:0000003) that does not have any fresh pollen cones (PPO:0001046).

pollen cones absent

OBO ID: PPO:0002646

IRI: http://purl.obolibrary.org/obo/PPO_0002646

Definition: A 'pollen cone presence' (PPO:0002049) trait that is a 'quality of' (RO:0000080) a 'whole plant' (PO:0000003) that does not have any pollen cones (PO:0005031).

cones absent

OBO ID: PPO:0002645

IRI: http://purl.obolibrary.org/obo/PPO_0002645

Definition: A 'cone presence' (PPO:0002048) trait that is a 'quality of' (RO:0000080) a 'whole plant' (PO:0000003) that does not have any cones (PO:0025083).

reproductive structures absent

OBO ID: PPO:0002622

IRI: http://purl.obolibrary.org/obo/PPO_0002622

Definition: A 'reproductive structure presence' (PPO:0002025) trait that is a 'quality of' (RO:0000080) a 'whole plant' (PO:0000003) that does not have any reproductive structures (PPO:0001023).

reproductive structures absent

OBO ID: PPO:0002622

IRI: http://purl.obolibrary.org/obo/PPO_0002622

Definition: A 'reproductive structure presence' (PPO:0002025) trait that is a 'quality of' (RO:0000080) a 'whole plant' (PO:0000003) that does not have any reproductive structures (PPO:0001023).

ripe fruits absent

OBO ID: PPO:0002644

IRI: http://purl.obolibrary.org/obo/PPO_0002644

Definition: A 'ripe fruit presence' (PPO:0002047) trait that is a 'quality of' (RO:0000080) a 'whole plant' (PO:0000003) that does not have any ripe fruits (PPO:0001045).

ripening fruits absent

OBO ID: PPO:0002642

IRI: http://purl.obolibrary.org/obo/PPO_0002642

Definition: A 'ripening fruit presence' (PPO:0002045) trait that is a 'quality of' (RO:0000080) a 'whole plant' (PO:0000003) that does not have any ripening fruits (PPO:0001043).

fruits absent

OBO ID: PPO:0002641

IRI: http://purl.obolibrary.org/obo/PPO_0002641

Definition: A 'fruit presence' (PPO:0002044) trait that is a 'quality of' (RO:0000080) a 'whole plant' (PO:0000003) that does not have any simple fruits or compound fruits (PPO:0001042).

reproductive structures absent

OBO ID: PPO:0002622

IRI: http://purl.obolibrary.org/obo/PPO_0002622

Definition: A 'reproductive structure presence' (PPO:0002025) trait that is a 'quality of' (RO:0000080) a 'whole plant' (PO:0000003) that does not have any reproductive structures (PPO:0001023).

ripe seed cones absent

OBO ID: PPO:0002654

IRI: http://purl.obolibrary.org/obo/PPO_0002654

Definition: A 'ripe seed cone presence' (PPO:0002057) trait that is a 'quality of' (RO:0000080) a 'whole plant' (PO:0000003) that does not have any ripe seed cones (PPO:0001052).

ripening seed cones absent

OBO ID: PPO:0002652

IRI: http://purl.obolibrary.org/obo/PPO_0002652

Definition: A 'ripening seed cone presence' (PPO:0002055) trait that is a 'quality of' (RO:0000080) a 'whole plant' (PO:0000003) that does not have any ripening seed cones (PPO:0001050).

fresh seed cones absent

OBO ID: PPO:0002651

IRI: http://purl.obolibrary.org/obo/PPO_0002651

Definition: A 'fresh seed cone presence' (PPO:0002054) trait that is a 'quality of' (RO:0000080) a 'whole plant' (PO:0000003) that does not have any fresh seed cones (PPO:0001049).

seed cones absent

OBO ID: PPO:0002650

IRI: http://purl.obolibrary.org/obo/PPO_0002650

Definition: A 'seed cone presence' (PPO:0002053) trait that is a 'quality of' (RO:0000080) a 'whole plant' (PO:0000003) that does not have any seed cones (PO:0005032).

cones absent

OBO ID: PPO:0002645

IRI: http://purl.obolibrary.org/obo/PPO_0002645

Definition: A 'cone presence' (PPO:0002048) trait that is a 'quality of' (RO:0000080) a 'whole plant' (PO:0000003) that does not have any cones (PO:0025083).

reproductive structures absent

OBO ID: PPO:0002622

IRI: http://purl.obolibrary.org/obo/PPO_0002622

Definition: A 'reproductive structure presence' (PPO:0002025) trait that is a 'quality of' (RO:0000080) a 'whole plant' (PO:0000003) that does not have any reproductive structures (PPO:0001023).

ripening fruits absent

OBO ID: PPO:0002642

IRI: http://purl.obolibrary.org/obo/PPO_0002642

Definition: A 'ripening fruit presence' (PPO:0002045) trait that is a 'quality of' (RO:0000080) a 'whole plant' (PO:0000003) that does not have any ripening fruits (PPO:0001043).

fruits absent

OBO ID: PPO:0002641

IRI: http://purl.obolibrary.org/obo/PPO_0002641

Definition: A 'fruit presence' (PPO:0002044) trait that is a 'quality of' (RO:0000080) a 'whole plant' (PO:0000003) that does not have any simple fruits or compound fruits (PPO:0001042).

reproductive structures absent

OBO ID: PPO:0002622

IRI: http://purl.obolibrary.org/obo/PPO_0002622

Definition: A 'reproductive structure presence' (PPO:0002025) trait that is a 'quality of' (RO:0000080) a 'whole plant' (PO:0000003) that does not have any reproductive structures (PPO:0001023).

ripening seed cones absent

OBO ID: PPO:0002652

IRI: http://purl.obolibrary.org/obo/PPO_0002652

Definition: A 'ripening seed cone presence' (PPO:0002055) trait that is a 'quality of' (RO:0000080) a 'whole plant' (PO:0000003) that does not have any ripening seed cones (PPO:0001050).

fresh seed cones absent

OBO ID: PPO:0002651

IRI: http://purl.obolibrary.org/obo/PPO_0002651

Definition: A 'fresh seed cone presence' (PPO:0002054) trait that is a 'quality of' (RO:0000080) a 'whole plant' (PO:0000003) that does not have any fresh seed cones (PPO:0001049).

seed cones absent

OBO ID: PPO:0002650

IRI: http://purl.obolibrary.org/obo/PPO_0002650

Definition: A 'seed cone presence' (PPO:0002053) trait that is a 'quality of' (RO:0000080) a 'whole plant' (PO:0000003) that does not have any seed cones (PO:0005032).

cones absent

OBO ID: PPO:0002645

IRI: http://purl.obolibrary.org/obo/PPO_0002645

Definition: A 'cone presence' (PPO:0002048) trait that is a 'quality of' (RO:0000080) a 'whole plant' (PO:0000003) that does not have any cones (PO:0025083).

reproductive structures absent

OBO ID: PPO:0002622

IRI: http://purl.obolibrary.org/obo/PPO_0002622

Definition: A 'reproductive structure presence' (PPO:0002025) trait that is a 'quality of' (RO:0000080) a 'whole plant' (PO:0000003) that does not have any reproductive structures (PPO:0001023).

seed cones absent

OBO ID: PPO:0002650

IRI: http://purl.obolibrary.org/obo/PPO_0002650

Definition: A 'seed cone presence' (PPO:0002053) trait that is a 'quality of' (RO:0000080) a 'whole plant' (PO:0000003) that does not have any seed cones (PO:0005032).

cones absent

OBO ID: PPO:0002645

IRI: http://purl.obolibrary.org/obo/PPO_0002645

Definition: A 'cone presence' (PPO:0002048) trait that is a 'quality of' (RO:0000080) a 'whole plant' (PO:0000003) that does not have any cones (PO:0025083).

reproductive structures absent

OBO ID: PPO:0002622

IRI: http://purl.obolibrary.org/obo/PPO_0002622

Definition: A 'reproductive structure presence' (PPO:0002025) trait that is a 'quality of' (RO:0000080) a 'whole plant' (PO:0000003) that does not have any reproductive structures (PPO:0001023).

seedling absent

OBO ID: PPO:0002604

IRI: http://purl.obolibrary.org/obo/PPO_0002604

Definition: An 'seedling presence' (PPO:0002007) trait that is a 'quality of' (RO:0000080) a 'whole plant' (PO:0000003) that is not a 'seedling' (PPO:0001007).

new shoot systems emerging from ground in first growth cycle absent

OBO ID: PPO:0002603

IRI: http://purl.obolibrary.org/obo/PPO_0002603

Definition: A 'new shoot system emerging from ground in first growth cycle presence' (PPO:0002006) trait that is a 'quality of' (RO:0000080) a 'whole plant' (PO:0000003) that does not have any new shoot systems emerging from ground in first growth cycle (PPO:0001006).

new shoot systems emerging from ground absent

OBO ID: PPO:0002602

IRI: http://purl.obolibrary.org/obo/PPO_0002602

Definition: A 'new shoot system emerging from ground presence' (PPO:0002005) trait that is a 'quality of' (RO:0000080) a 'whole plant' (PO:0000003) that does not have any new shoot systems emerging from ground (PPO:0001005).

new shoot system absent

OBO ID: PPO:0002600

IRI: http://purl.obolibrary.org/obo/PPO_0002600

Definition: A 'new shoot system presence' (PPO:0002003) trait that is a 'quality of' (RO:0000080) a 'whole plant' (PO:0000003) that does not have any new shoot systems (PPO:0001003).

senesced floral structures absent

OBO ID: PPO:0002628

IRI: http://purl.obolibrary.org/obo/PPO_0002628

Definition: A 'senesced floral structure presence' (PPO:0002031) trait that is a 'quality of' (RO:0000080) a 'whole plant' (PO:0000003) that does not have any senesced floral structures (PPO:0001030).

senesced flowers absent

OBO ID: PPO:0002634

IRI: http://purl.obolibrary.org/obo/PPO_0002634

Definition: A 'senesced flower presence' (PPO:0002037) trait that is a 'quality of' (RO:0000080) a 'whole plant' (PO:0000003) that does not have any senesced flowers (PPO:0001035).

flowers absent

OBO ID: PPO:0002629

IRI: http://purl.obolibrary.org/obo/PPO_0002629

Definition: A 'flower presence' (PPO:0002032) trait that is a 'quality of' (RO:0000080) a 'whole plant' (PO:0000003) that does not have any PO:flowers (PO:0009046).

floral structures absent

OBO ID: PPO:0002623

IRI: http://purl.obolibrary.org/obo/PPO_0002623

Definition: A 'floral structure presence' (PPO:0002026) trait that is a 'quality of' (RO:0000080) a 'whole plant' (PO:0000003) that does not have any floral structures (PPO:0001025).

reproductive structures absent

OBO ID: PPO:0002622

IRI: http://purl.obolibrary.org/obo/PPO_0002622

Definition: A 'reproductive structure presence' (PPO:0002025) trait that is a 'quality of' (RO:0000080) a 'whole plant' (PO:0000003) that does not have any reproductive structures (PPO:0001023).

senesced flower heads absent

OBO ID: PPO:0002640

IRI: http://purl.obolibrary.org/obo/PPO_0002640

Definition: A 'senesced flower head presence' (PPO:0002043) trait that is a 'quality of' (RO:0000080) a 'whole plant' (PO:0000003) that does not have any senesced flower heads (PPO:0001040).

flower heads absent

OBO ID: PPO:0002635

IRI: http://purl.obolibrary.org/obo/PPO_0002635

Definition: A 'flower head presence' (PPO:0002038) trait that is a 'quality of' (RO:0000080) a 'whole plant' (PO:0000003) that does not have any PO:inflorescences (PO:0009049).

floral structures absent

OBO ID: PPO:0002623

IRI: http://purl.obolibrary.org/obo/PPO_0002623

Definition: A 'floral structure presence' (PPO:0002026) trait that is a 'quality of' (RO:0000080) a 'whole plant' (PO:0000003) that does not have any floral structures (PPO:0001025).

reproductive structures absent

OBO ID: PPO:0002622

IRI: http://purl.obolibrary.org/obo/PPO_0002622

Definition: A 'reproductive structure presence' (PPO:0002025) trait that is a 'quality of' (RO:0000080) a 'whole plant' (PO:0000003) that does not have any reproductive structures (PPO:0001023).

senesced flowers absent

OBO ID: PPO:0002634

IRI: http://purl.obolibrary.org/obo/PPO_0002634

Definition: A 'senesced flower presence' (PPO:0002037) trait that is a 'quality of' (RO:0000080) a 'whole plant' (PO:0000003) that does not have any senesced flowers (PPO:0001035).

flowers absent

OBO ID: PPO:0002629

IRI: http://purl.obolibrary.org/obo/PPO_0002629

Definition: A 'flower presence' (PPO:0002032) trait that is a 'quality of' (RO:0000080) a 'whole plant' (PO:0000003) that does not have any PO:flowers (PO:0009046).

floral structures absent

OBO ID: PPO:0002623

IRI: http://purl.obolibrary.org/obo/PPO_0002623

Definition: A 'floral structure presence' (PPO:0002026) trait that is a 'quality of' (RO:0000080) a 'whole plant' (PO:0000003) that does not have any floral structures (PPO:0001025).

reproductive structures absent

OBO ID: PPO:0002622

IRI: http://purl.obolibrary.org/obo/PPO_0002622

Definition: A 'reproductive structure presence' (PPO:0002025) trait that is a 'quality of' (RO:0000080) a 'whole plant' (PO:0000003) that does not have any reproductive structures (PPO:0001023).

senescing true leaves absent

OBO ID: PPO:0002616

IRI: http://purl.obolibrary.org/obo/PPO_0002616

Definition: A 'senescing true leaf presence' (PPO:0002019) trait that is a 'quality of' (RO:0000080) a 'whole plant' (PO:0000003) that does not have any senescing true leaves (PPO:0001017).

unfolded true leaves absent

OBO ID: PPO:0002614

IRI: http://purl.obolibrary.org/obo/PPO_0002614

Definition: An 'unfolded true leaf presence' (PPO:0002017) trait that is a 'quality of' (RO:0000080) a 'whole plant' (PO:0000003) that does not have any unfolded true leaves (PPO:0001015).

true leaves absent

OBO ID: PPO:0002612

IRI: http://purl.obolibrary.org/obo/PPO_0002612

Definition: A 'true leaf presence' (PPO:0002015) trait that is a 'quality of' (RO:0000080) a 'whole plant' (PO:0000003) that does not have any true leaves (PPO:0001013).

vascular leaves absent

OBO ID: PPO:0002611

IRI: http://purl.obolibrary.org/obo/PPO_0002611

Definition: A 'vascular leaf presence' (PPO:0002014) trait that is a 'quality of' (RO:0000080) a 'whole plant' (PO:0000003) that does not have any vascular leaves (PO:0009025).

swelling leaf buds absent

OBO ID: PPO:0002609

IRI: http://purl.obolibrary.org/obo/PPO_0002609

Definition: A 'swelling leaf bud presence' (PPO:0002012) trait that is a 'quality of' (RO:0000080) a 'whole plant' (PO:0000003) that does not have any swelling leaf buds (PPO:0001011).

non-dormant leaf buds absent

OBO ID: PPO:0002608

IRI: http://purl.obolibrary.org/obo/PPO_0002608

Definition: A 'non-dormant leaf bud presence' (PPO:0002011) trait that is a 'quality of' (RO:0000080) a 'whole plant' (PO:0000003) that does not have any non-dormant leaf buds (PPO:0001010).

leaf buds absent

OBO ID: PPO:0002606

IRI: http://purl.obolibrary.org/obo/PPO_0002606

> > Definition: A 'leaf bud presence' (PPO:0002009) trait that is a 'quality of' (RO:0000080) a 'whole plant' (PO:0000003) that does not have any vegetative buds (PO:0000058).

true leaves absent

OBO ID: PPO:0002612

IRI: http://purl.obolibrary.org/obo/PPO_0002612

Definition: A 'true leaf presence' (PPO:0002015) trait that is a 'quality of' (RO:0000080) a 'whole plant' (PO:0000003) that does not have any true leaves (PPO:0001013).

> #### vascular leaves absent
>
> OBO ID: PPO:0002611
>
> IRI: http://purl.obolibrary.org/obo/PPO_0002611
>
> Definition: A 'vascular leaf presence' (PPO:0002014) trait that is a 'quality of' (RO:0000080) a 'whole plant' (PO:0000003) that does not have any vascular leaves (PO:0009025).

unfolded true leaves absent

OBO ID: PPO:0002614

IRI: http://purl.obolibrary.org/obo/PPO_0002614

Definition: An 'unfolded true leaf presence' (PPO:0002017) trait that is a 'quality of' (RO:0000080) a 'whole plant' (PO:0000003) that does not have any unfolded true leaves (PPO:0001015).

> #### true leaves absent
>
> OBO ID: PPO:0002612
>
> IRI: http://purl.obolibrary.org/obo/PPO_0002612
>
> Definition: A 'true leaf presence' (PPO:0002015) trait that is a 'quality of' (RO:0000080) a 'whole plant' (PO:0000003) that does not have any true leaves (PPO:0001013).
>
> > ##### vascular leaves absent
> >
> > OBO ID: PPO:0002611
> >
> > IRI: http://purl.obolibrary.org/obo/PPO_0002611
> >
> > Definition: A 'vascular leaf presence' (PPO:0002014) trait that is a 'quality of' (RO:0000080) a 'whole plant' (PO:0000003) that does not have any vascular leaves (PO:0009025).

unfolding true leaves absent

OBO ID: PPO:0002613

IRI: http://purl.obolibrary.org/obo/PPO_0002613

Definition: An 'unfolding true leaf presence' (PPO:0002016) trait that is a 'quality of' (RO:0000080) a 'whole plant' (PO:0000003) that does not have any unfolding true leaves (PPO:0001014).

expanding true leaves absent

OBO ID: PPO:0002621

IRI: http://purl.obolibrary.org/obo/PPO_0002621

Definition: An 'expanding true leaf presence' (PPO:0002024) trait that is a 'quality of' (RO:0000080) a 'whole plant' (PO:0000003) that does not have any expanding true leaves (PPO:0001022).

true leaves absent

OBO ID: PPO:0002612

IRI: http://purl.obolibrary.org/obo/PPO_0002612

Definition: A 'true leaf presence' (PPO:0002015) trait that is a 'quality of' (RO:0000080) a 'whole plant' (PO:0000003) that does not have any true leaves (PPO:0001013).

vascular leaves absent

OBO ID: PPO:0002611

IRI: http://purl.obolibrary.org/obo/PPO_0002611

Definition: A 'vascular leaf presence' (PPO:0002014) trait that is a 'quality of' (RO:0000080) a 'whole plant' (PO:0000003) that does not have any vascular leaves (PO:0009025).

unopened floral structures absent

OBO ID: PPO:0002625

IRI: http://purl.obolibrary.org/obo/PPO_0002625

Definition: An 'unopened floral structure presence' (PPO:0002028) trait that is a 'quality of' (RO:0000080) a 'whole plant' (PO:0000003) that does not have any unopened floral structures (PPO:0001027).

non-senesced floral structures absent

OBO ID: PPO:0002624

IRI: http://purl.obolibrary.org/obo/PPO_0002624

Definition: A 'non-senesced floral structure presence' (PPO:0002027) trait that is a 'quality of' (RO:0000080) a 'whole plant' (PO:0000003) that does not have any non-senesced floral structures (PPO:0001026).

floral structures absent

OBO ID: PPO:0002623

IRI: http://purl.obolibrary.org/obo/PPO_0002623

Definition: A 'floral structure presence' (PPO:0002026) trait that is a 'quality of' (RO:0000080) a 'whole plant' (PO:0000003) that does not have any floral structures (PPO:0001025).

reproductive structures absent

OBO ID: PPO:0002622

IRI: http://purl.obolibrary.org/obo/PPO_0002622

Definition: A 'reproductive structure presence' (PPO:0002025) trait that is a 'quality of' (RO:0000080) a 'whole plant' (PO:0000003) that does not have any reproductive structures (PPO:0001023).

unopened flower heads absent

OBO ID: PPO:0002637

IRI: http://purl.obolibrary.org/obo/PPO_0002637

Definition: An 'unopened flower head presence' (PPO:0002040) trait that is a 'quality of' (RO:0000080) a 'whole plant' (PO:0000003) that does not have any unopened flower heads (PPO:0001037).

non-senesced flower heads absent

OBO ID: PPO:0002636

IRI: http://purl.obolibrary.org/obo/PPO_0002636

Definition: A 'non-senesced flower head presence' (PPO:0002039) trait that is a 'quality of' (RO:0000080) a 'whole plant' (PO:0000003) that does not have any non-senesced flower heads (PPO:0001036).

flower heads absent

OBO ID: PPO:0002635

IRI: http://purl.obolibrary.org/obo/PPO_0002635

Definition: A 'flower head presence' (PPO:0002038) trait that is a 'quality of' (RO:0000080) a 'whole plant' (PO:0000003) that does not have any PO:inflorescences (PO:0009049).

floral structures absent

OBO ID: PPO:0002623

IRI: http://purl.obolibrary.org/obo/PPO_0002623

Definition: A 'floral structure presence' (PPO:0002026) trait that is a 'quality of' (RO:0000080) a 'whole plant' (PO:0000003) that does not have any floral structures (PPO:0001025).

reproductive structures absent

OBO ID: PPO:0002622

IRI: http://purl.obolibrary.org/obo/PPO_0002622

Definition: A 'reproductive structure presence' (PPO:0002025) trait that is a 'quality of' (RO:0000080) a 'whole plant' (PO:0000003) that does not have any reproductive structures (PPO:0001023).

unopened flowers absent

OBO ID: PPO:0002631

IRI: http://purl.obolibrary.org/obo/PPO_0002631

Definition: An 'unopened flower presence' (PPO:0002034) trait that is a 'quality of' (RO:0000080) a 'whole plant' (PO:0000003) that does not have any unopened flowers (PPO:0001032).

non-senesced flowers absent

OBO ID: PPO:0002630

IRI: http://purl.obolibrary.org/obo/PPO_0002630

Definition: A 'non-senesced flower presence' (PPO:0002033) trait that is a 'quality of' (RO:0000080) a 'whole plant' (PO:0000003) that does not have any non-senesced flowers (PPO:0001031).

flowers absent

OBO ID: PPO:0002629

IRI: http://purl.obolibrary.org/obo/PPO_0002629

Definition: A 'flower presence' (PPO:0002032) trait that is a 'quality of' (RO:0000080) a 'whole plant' (PO:0000003) that does not have any PO:flowers (PO:0009046).

floral structures absent

OBO ID: PPO:0002623

IRI: http://purl.obolibrary.org/obo/PPO_0002623

Definition: A 'floral structure presence' (PPO:0002026) trait that is a 'quality of' (RO:0000080) a 'whole plant' (PO:0000003) that does not have any floral structures (PPO:0001025).

reproductive structures absent

OBO ID: PPO:0002622

IRI: http://purl.obolibrary.org/obo/PPO_0002622

Definition: A 'reproductive structure presence' (PPO:0002025) trait that is a 'quality of' (RO:0000080) a 'whole plant' (PO:0000003) that does not have any reproductive structures (PPO:0001023).

unripe fruits absent

OBO ID: PPO:0002643

IRI: http://purl.obolibrary.org/obo/PPO_0002643

Definition: An 'unripe fruit presence' (PPO:0002046) trait that is a 'quality of' (RO:0000080) a 'whole plant' (PO:0000003) that does not have any unripe fruits (PPO:0001044).

ripening fruits absent

OBO ID: PPO:0002642

IRI: http://purl.obolibrary.org/obo/PPO_0002642

Definition: A 'ripening fruit presence' (PPO:0002045) trait that is a 'quality of' (RO:0000080) a 'whole plant' (PO:0000003) that does not have any ripening fruits (PPO:0001043).

fruits absent

OBO ID: PPO:0002641

IRI: http://purl.obolibrary.org/obo/PPO_0002641

Definition: A 'fruit presence' (PPO:0002044) trait that is a 'quality of' (RO:0000080) a 'whole plant' (PO:0000003) that does not have any simple fruits or compound fruits (PPO:0001042).

reproductive structures absent

OBO ID: PPO:0002622

IRI: http://purl.obolibrary.org/obo/PPO_0002622

Definition: A 'reproductive structure presence' (PPO:0002025) trait that is a 'quality of' (RO:0000080) a 'whole plant' (PO:0000003) that does not have any reproductive structures (PPO:0001023).

unripe seed cones absent

OBO ID: PPO:0002653

IRI: http://purl.obolibrary.org/obo/PPO_0002653

Definition: An 'unripe seed cone presence' (PPO:0002056) trait that is a 'quality of' (RO:0000080) a 'whole plant' (PO:0000003) that does not have any unripe seed cones (PPO:0001051).

ripening seed cones absent

OBO ID: PPO:0002652

IRI: http://purl.obolibrary.org/obo/PPO_0002652

Definition: A 'ripening seed cone presence' (PPO:0002055) trait that is a 'quality of' (RO:0000080) a 'whole plant' (PO:0000003) that does not have any ripening seed cones (PPO:0001050).

fresh seed cones absent

OBO ID: PPO:0002651

IRI: http://purl.obolibrary.org/obo/PPO_0002651

Definition: A 'fresh seed cone presence' (PPO:0002054) trait that is a 'quality of' (RO:0000080) a 'whole plant' (PO:0000003) that does not have any fresh seed cones (PPO:0001049).

seed cones absent

OBO ID: PPO:0002650

IRI: http://purl.obolibrary.org/obo/PPO_0002650

Definition: A 'seed cone presence' (PPO:0002053) trait that is a 'quality of' (RO:0000080) a 'whole plant' (PO:0000003) that does not have any seed cones (PO:0005032).

cones absent

OBO ID: PPO:0002645

IRI: http://purl.obolibrary.org/obo/PPO_0002645

Definition: A 'cone presence' (PPO:0002048) trait that is a 'quality of' (RO:0000080) a 'whole plant' (PO:0000003) that does not have any cones (PO:0025083).

reproductive structures absent

OBO ID: PPO:0002622

IRI: http://purl.obolibrary.org/obo/PPO_0002622

Definition: A 'reproductive structure presence' (PPO:0002025) trait that is a 'quality of' (RO:0000080) a 'whole plant' (PO:0000003) that does not have any reproductive structures (PPO:0001023).

vascular leaves absent

OBO ID: PPO:0002611

IRI: http://purl.obolibrary.org/obo/PPO_0002611

Definition: A 'vascular leaf presence' (PPO:0002014) trait that is a 'quality of' (RO:0000080) a 'whole plant' (PO:0000003) that does not have any vascular leaves (PO:0009025).

abscised cones or seeds absent

OBO ID: PPO:0002658

IRI: http://purl.obolibrary.org/obo/PPO_0002658

Definition: An 'abscised cone or seed presence' (PPO:0002060) trait that is a 'quality of' (RO:0000080) a 'whole plant' (PO:0000003) from which no ripe seed cones (PPO:0001052) have been abscised or removed by herbivores and that does not have any ripe seed cones that have 'abscised' (PPO:0000005) any mature seeds (PPO:0001024).

abscised plant structures absent

OBO ID: PPO:0002655

> IRI: http://purl.obolibrary.org/obo/PPO_0002655
>
> Definition: An 'abscised plant structure presence' (PPO:0002002) trait that is a 'quality of' (RO:0000080) a 'whole plant' (PO:0000003) from which none of some 'plant structure' (PO:0009011) have been abscised or removed.

abscised fruits or seeds absent

OBO ID: PPO:0002657

IRI: http://purl.obolibrary.org/obo/PPO_0002657

Definition: An 'abscised fruit or seed presence' (PPO:0002059) trait that is a 'quality of' (RO:0000080) a 'whole plant' (PO:0000003) from which no ripe fruits (PPO:0001045) have been abscised or removed by herbivores and that does not have any ripe fruits that have 'abscised' (PPO:0000005) any mature seeds (PPO:0001024).

> ### abscised plant structures absent
>
> OBO ID: PPO:0002655
>
> IRI: http://purl.obolibrary.org/obo/PPO_0002655
>
> Definition: An 'abscised plant structure presence' (PPO:0002002) trait that is a 'quality of' (RO:0000080) a 'whole plant' (PO:0000003) from which none of some 'plant structure' (PO:0009011) have been abscised or removed.

abscised leaves absent

OBO ID: PPO:0002656

IRI: http://purl.obolibrary.org/obo/PPO_0002656

Definition: An 'abscised leaf presence' (PPO:0002058) trait that is a 'quality of' (RO:0000080) a 'whole plant' (PO:0000003) from which no true leaves (PPO:0001013) have been 'abscised' (PPO:0000005).

> ### abscised plant structures absent
>
> OBO ID: PPO:0002655
>
> IRI: http://purl.obolibrary.org/obo/PPO_0002655
>
> Definition: An 'abscised plant structure presence' (PPO:0002002) trait that is a 'quality of' (RO:0000080) a 'whole plant' (PO:0000003) from which none of some 'plant structure' (PO:0009011) have been abscised or removed.

Plant structures

In the PPO, plant structures are used primarily to provide precise definitions of phenological traits. Most users will probably not need to use the plant structure classes directly, but they are included here for reference. Many of the plant structures used in the PPO are defined in the Plant Ontology (PO), which includes many more plant structures that are not used by the PPO. Here, we only include the PO plant structures that are most relevant to users of the PPO.

plant structure

OBO ID: PO:0009011

IRI: http://purl.obolibrary.org/obo/PO_0009011

Definition: An anatomical structure that is or was part of a plant, or was derived from a part of a plant.

Comment: 'Part' includes both proper parts and the whole plant. CARO:0000003 anatomical structure is defined as: Material anatomical entity that has inherent 3D shape and is generated by coordinated expression of the organism's own genome.

reproductive structure

OBO ID: PPO:0001023

IRI: http://purl.obolibrary.org/obo/PPO_0001023

Definition: A 'plant structure' (PO:0009011) that is either a 'reproductive shoot system' (PO:0025082) or a 'fruit' (PO:0009001).

reproductive shoot system

OBO ID: PO:0025082

IRI: http://purl.obolibrary.org/obo/PO_0025082

Definition: A shoot system (PO:0009006) in the sporophytic phase that has as part at least one sporangium (PO:0025094).

floral structure

OBO ID: PPO:0001025

IRI: http://purl.obolibrary.org/obo/PPO_0001025

flower

OBO ID: PO:0009046

IRI: http://purl.obolibrary.org/obo/PO_0009046

Definition: A determinate reproductive shoot system (PO:0025082) that has as part at least one carpel (PO:0009030) or at least one stamen (PO:0009029) and does not contain any other determinate shoot system (PO:0009006) as a part.

Comment: The characteristic reproductive structure of angiosperms. May have as part one or more petals, sepals or tepals. May contain one or more pistillode (PO:0009078), staminode (PO:0009077) or other aborted organs that don't show up in mature form.

non-senesced flower

OBO ID: PPO:0001031

IRI: http://purl.obolibrary.org/obo/PPO_0001031

Definition: A 'flower' (PO:0009046) in which all the petaloid floral organs (PO:0025395) have not completed 'floral organ senescence' (GO:0080187). Petaloid floral organs generally are the petals, sepal, or tepals, but may also be floral bracts or petaloid anthers (PO:0009066). For scoring phenology in grasses, petaloid floral organs can include 'lemma' (PO:0009037), 'palea' (PO:0009038) or 'glume' (PO:0009039), even though a 'glume' (PO:0009039) is not strictly a 'floral organ' (PO:0025395), because it encloses an 'inflorescence' (PO:0009049).

open flower

OBO ID: PPO:0001033

IRI: http://purl.obolibrary.org/obo/PPO_0001033

Definition: A 'non-senesced flower' (PPO:0001031) that is in the 'open flower stage' (PPO:0007010).

pollen-releasing flower

OBO ID: PPO:0001034

IRI: http://purl.obolibrary.org/obo/PPO_0001034

Definition: An 'open flower' (PPO:0001033) that is in the 'pollen-releasing flower stage' (PPO:0007011).

unopened flower

OBO ID: PPO:0001032

IRI: http://purl.obolibrary.org/obo/PPO_0001032

Definition: A 'non-senesced flower' (PPO:0001031) that is in the 'unopened flower stage' (PPO:0007009).

senesced flower

OBO ID: PPO:0001035

IRI: http://purl.obolibrary.org/obo/PPO_0001035

Definition: A 'flower' (PO:0009046) in which all of the petaloid floral organs (PO:0025395) have completed 'floral organ senescence' (GO:0080187). Petaloid floral organs generally

are the petals, sepal, or tepals, but may also be floral bracts or petaloid anthers (PO:0009066). For scoring phenology in grasses, petaloid floral organs can include 'lemma' (PO:0009037), 'palea' (PO:0009038) or 'glume' (PO:0009039), even though a 'glume' (PO:0009039) is not strictly a 'floral organ' (PO:0025395), because it encloses an 'inflorescence' (PO:0009049).

spikelet floret

OBO ID: PO:0009082

IRI: http://purl.obolibrary.org/obo/PO_0009082

Definition: A small flower (PO:0009046) that is part of a spikelet (PO:0009051).

Comment: A spikelet floret (PO:0009082) is enclosed by one or both the flower bracts (PO:0009034); lemma (PO:0009037) and palea (PO:0009038) and also includes a lodicule (PO:0009036). To describe a floret of the Asteraceae, use ray flower (PO:0025331) or disk flower (PO:0025332). Found in Poaceae, Cyperaceae and other Poales.

inflorescence

OBO ID: PO:0009049

IRI: http://purl.obolibrary.org/obo/PO_0009049

Definition: A reproductive shoot system (PO:0025082) that has as parts all of the shoot axes (PO:0025029) distal to the most distal foliage leaf (PO:0009025) of a shoot axis and all of the flowers (PO:0009046) borne by those axes. Must have two or more flowers as parts.

Comment: Some plants have only solitary flowers, e.g. *Magnolia*.

non-senesced flower head

OBO ID: PPO:0001036

IRI: http://purl.obolibrary.org/obo/PPO_0001036

Definition: An 'inflorescence' (PO:0009049) with at least one 'non-senesced flower' (PPO:0001031). In the case of an 'unopened flower head' (PPO:0001037), the non-senesced flower(s) might not be visible.

open flower head

OBO ID: PPO:0001038

IRI: http://purl.obolibrary.org/obo/PPO_0001038

Definition: A 'non-senesced flower head' (PPO:0001036) with at least one 'open flower' (PPO:0001033).

pollen-releasing flower head

OBO ID: PPO:0001039

IRI: http://purl.obolibrary.org/obo/PPO_0001039

Definition: An 'open flower head' (PPO:0001038) with at least one 'pollen-releasing flower' (PPO:0001034).

unopened flower head

OBO ID: PPO:0001037

IRI: http://purl.obolibrary.org/obo/PPO_0001037

Definition: A 'non-senesced flower head' (PPO:0001036) in which all flowers are unopened flowers (PPO:0001032). The unopened flowers might not yet be visible.

senesced flower head

OBO ID: PPO:0001040

IRI: http://purl.obolibrary.org/obo/PPO_0001040

Definition: An 'inflorescence' (PO:0009049) on which there are no remaining non-senesced flowers (PPO:0001031).

non-senesced floral structure

OBO ID: PPO:0001026

IRI: http://purl.obolibrary.org/obo/PPO_0001026

open floral structure

OBO ID: PPO:0001028

IRI: http://purl.obolibrary.org/obo/PPO_0001028

pollen-releasing floral structure

OBO ID: PPO:0001029

IRI: http://purl.obolibrary.org/obo/PPO_0001029

unopened floral structure

OBO ID: PPO:0001027

IRI: http://purl.obolibrary.org/obo/PPO_0001027

senesced floral structure

OBO ID: PPO:0001030

IRI: http://purl.obolibrary.org/obo/PPO_0001030

Definition: A 'floral structure' (PPO:0001025) that has completed 'floral organ senescence' (GO:0080187).

flower

OBO ID: PO:0009046

IRI: http://purl.obolibrary.org/obo/PO_0009046

Definition: A determinate reproductive shoot system (PO:0025082) that has as part at least one carpel (PO:0009030) or at least one stamen (PO:0009029) and does not contain any other determinate shoot system (PO:0009006) as a part.

Comment: The characteristic reproductive structure of angiosperms. May have as part one or more petals, sepals or tepals. May contain one or more pistillode (PO:0009078), staminode (PO:0009077) or other aborted organs that don't show up in mature form.

non-senesced flower

OBO ID: PPO:0001031

IRI: http://purl.obolibrary.org/obo/PPO_0001031

Definition: A 'flower' (PO:0009046) in which all the petaloid floral organs (PO:0025395) have not completed 'floral organ senescence' (GO:0080187). Petaloid floral organs generally are the petals, sepal, or tepals, but may also be floral bracts or petaloid anthers (PO:0009066). For scoring phenology in grasses, petaloid floral organs can include 'lemma' (PO:0009037), 'palea' (PO:0009038) or 'glume' (PO:0009039), even though a 'glume' (PO:0009039) is not strictly a 'floral organ' (PO:0025395), because it encloses an 'inflorescence' (PO:0009049).

open flower

OBO ID: PPO:0001033

IRI: http://purl.obolibrary.org/obo/PPO_0001033

Definition: A 'non-senesced flower' (PPO:0001031) that is in the 'open flower stage' (PPO:0007010).

pollen-releasing flower

OBO ID: PPO:0001034

IRI: http://purl.obolibrary.org/obo/PPO_0001034

Definition: An 'open flower' (PPO:0001033) that is in the 'pollen-releasing flower stage' (PPO:0007011).

unopened flower

OBO ID: PPO:0001032

IRI: http://purl.obolibrary.org/obo/PPO_0001032

Definition: A 'non-senesced flower' (PPO:0001031) that is in the 'unopened flower stage' (PPO:0007009).

senesced flower

OBO ID: PPO:0001035

IRI: http://purl.obolibrary.org/obo/PPO_0001035

Definition: A 'flower' (PO:0009046) in which all of the petaloid floral organs (PO:0025395) have completed 'floral organ senescence' (GO:0080187). Petaloid floral organs generally are the petals, sepal, or tepals, but may also be floral bracts or petaloid anthers (PO:0009066). For scoring phenology in grasses, petaloid floral organs can include 'lemma' (PO:0009037), 'palea' (PO:0009038) or 'glume' (PO:0009039), even though a 'glume' (PO:0009039) is not strictly a 'floral organ' (PO:0025395), because it encloses an 'inflorescence' (PO:0009049).

spikelet floret

OBO ID: PO:0009082

IRI: http://purl.obolibrary.org/obo/PO_0009082

Definition: A small flower (PO:0009046) that is part of a spikelet (PO:0009051).

Comment: A spikelet floret (PO:0009082) is enclosed by one or both the flower bracts (PO:0009034); lemma (PO:0009037) and palea (PO:0009038) and also includes a lodicule (PO:0009036). To describe a floret of the Asteraceae, use ray flower (PO:0025331) or disk flower (PO:0025332). Found in Poaceae, Cyperaceae and other Poales.

inflorescence

OBO ID: PO:0009049

IRI: http://purl.obolibrary.org/obo/PO_0009049

Definition: A reproductive shoot system (PO:0025082) that has as parts all of the shoot axes (PO:0025029) distal to the most distal foliage leaf (PO:0009025) of a shoot axis and all of the flowers (PO:0009046) borne by those axes. Must have two or more flowers as parts.

Comment: Some plants have only solitary flowers, e.g. *Magnolia*.

non-senesced flower head

OBO ID: PPO:0001036

IRI: http://purl.obolibrary.org/obo/PPO_0001036

Definition: An 'inflorescence' (PO:0009049) with at least one 'non-senesced flower' (PPO:0001031). In the case of an 'unopened flower head' (PPO:0001037), the non-senesced flower(s) might not be visible.

open flower head

OBO ID: PPO:0001038

IRI: http://purl.obolibrary.org/obo/PPO_0001038

Definition: A 'non-senesced flower head' (PPO:0001036) with at least one 'open flower' (PPO:0001033).

pollen-releasing flower head

OBO ID: PPO:0001039

IRI: http://purl.obolibrary.org/obo/PPO_0001039

Definition: An 'open flower head' (PPO:0001038) with at least one 'pollen-releasing flower' (PPO:0001034).

unopened flower head

OBO ID: PPO:0001037

IRI: http://purl.obolibrary.org/obo/PPO_0001037

Definition: A 'non-senesced flower head' (PPO:0001036) in which all flowers are unopened flowers (PPO:0001032). The unopened flowers might not yet be visible.

senesced flower head

OBO ID: PPO:0001040

IRI: http://purl.obolibrary.org/obo/PPO_0001040

Definition: An 'inflorescence' (PO:0009049) on which there are no remaining non-senesced flowers (PPO:0001031).

reproductive bud

OBO ID: PO:0025084

IRI: http://purl.obolibrary.org/obo/PO_0025084

Definition: A bud that develops into a reproductive shoot system.

flower bud

OBO ID: PO:0000056

IRI: http://purl.obolibrary.org/obo/PO_0000056

Definition: A bud that develops into a flower.

inflorescence bud

OBO ID: PO:0000057

IRI: http://purl.obolibrary.org/obo/PO_0000057

Definition: A bud that develops into an inflorescence.

spikelet

OBO ID: PO:0009051

IRI: http://purl.obolibrary.org/obo/PO_0009051

Definition: A reproductive shoot system (PO:0025082) that is the ultimate and congested inflorescence branch (PO:0009081) of the grasses.

Comment: It consists of one to many closely-packed flowers and associated glumes etc.

strobilus

OBO ID: PO:0025083

IRI: http://purl.obolibrary.org/obo/PO_0025083

Definition: A reproductive shoot system (PO:0025082) consisting of a number of sporophylls (PO:0009026) or plant ovule (PO:0020003)-bearing modified leaves (PO:0025034) grouped terminally on a stem (PO:0009047).

Comment: May be simple or compound. Found in lycophytes such as Selaginella and Lycopodium, sphenophytes such as Equisetum, gymnosperms such as cycad, conifers, gnetophytes and the pollen producing structures of Gingko. The term cone is often used for strobili that are woody. Flowers are sometimes

considered bisexual strobili. Some flowering plants such as Alnus have inflorescences called catkins that resemble strobili, but are not.

megasporangiate strobilus

OBO ID: PO:0005032

IRI: http://purl.obolibrary.org/obo/PO_0005032

Definition: A strobilus (PO:0025083) that bears only plant ovules (PO:0020003).

fresh seed cone

OBO ID: PPO:0001049

IRI: http://purl.obolibrary.org/obo/PPO_0001049

Definition: A 'megasporangiate strobilus' (PO:0005032) that has not yet been abscised and has not yet releases all of its seeds.

ripening seed cone

OBO ID: PPO:0001050

IRI: http://purl.obolibrary.org/obo/PPO_0001050

Definition: A 'fresh seed cone' (PPO:0001049) that is in the 'megasporangiate strobilus ripening stage' (PPO:0007017).

ripe seed cone

OBO ID: PPO:0001052

IRI: http://purl.obolibrary.org/obo/PPO_0001052

Definition: A 'ripening seed cone' (PPO:0001050) that is in the 'megasporangiate strobilus ripe stage' (PPO:0007019).

unripe seed cone

OBO ID: PPO:0001051

IRI: http://purl.obolibrary.org/obo/PPO_0001051

Definition: A 'ripening seed cone' (PPO:0001050) that is in the 'megasporangiate strobilus unripe stage' (PPO:0007018).

microsporangiate strobilus

OBO ID: PO:0005031

IRI: http://purl.obolibrary.org/obo/PO_0005031

Definition: A strobilus (PO:0025083) that bears only pollen (PO:0025281).

fresh pollen cone

OBO ID: PPO:0001046

IRI: http://purl.obolibrary.org/obo/PPO_0001046

Definition: A 'microsporangiate strobilus' (PO:0005031) that has not yet completed the 'pollen-releasing strobilus stage' (PPO:0007016).

open pollen cone

OBO ID: PPO:0001047

IRI: http://purl.obolibrary.org/obo/PPO_0001047

Definition: A 'fresh pollen cone' (PPO:0001046) that is in the 'open microsporangiate strobilus stage' (PPO:0007015).

pollen-releasing pollen cone

OBO ID: PPO:0001048

IRI: http://purl.obolibrary.org/obo/PPO_0001048

Definition: A 'open pollen cone' (PPO:0001047) that is in the 'pollen-releasing strobilus stage' (PPO:0007016).

simple fruit or compound fruit

OBO ID: PPO:0001042

IRI: http://purl.obolibrary.org/obo/PPO_0001042

compound fruit

OBO ID: PPO:0001041

IRI: http://purl.obolibrary.org/obo/PPO_0001041

fruit

OBO ID: PO:0009001

IRI: http://purl.obolibrary.org/obo/PO_0009001

Definition: A multi-tissue plant structure (PO:0025496) that develops from a gynoecium (PO:0009062), or a single carpel (PO:0009030), and at maturity may have as parts one or more seeds (PO:0009010).

Comment: A fruit (PO:0009001) may contain additional plant structures (PO:0009011) that were part of a flower (PO:0009046) and mature along with the gynoecium, such as a receptacle (PO:0009064). A fruit may develop without fertilization in cases of parthenocarpy, apomixis, or other hormone-induced conditions and may not always contain seeds (PO:0009010). When annotating to fruit (PO:0009001) that are referred to as ‘aggregate’, ‘multiple’, or ‘compound’, please annotate directly to the appropriate plant structure, such as receptacle, hypanthium (PO:0009065) or infructescence (PO:0006342). Fruits only occur in angiosperms.

ripening fruit

OBO ID: PPO:0001043

IRI: http://purl.obolibrary.org/obo/PPO_0001043

Definition: A 'simple fruit or compound fruit' (PPO:0001042) that is in the PO:'fruit ripening stage' (PO:0025502).

ripe fruit

OBO ID: PPO:0001045

IRI: http://purl.obolibrary.org/obo/PPO_0001045

Definition: A 'ripening fruit' (PPO:0001043) that is in the 'fruit ripe stage' (PPO:0007013).

unripe fruit

OBO ID: PPO:0001044

IRI: http://purl.obolibrary.org/obo/PPO_0001044

Definition: A 'ripening fruit' (PPO:0001043) that is in the 'fruit unripe stage' (PPO:0007012).

bud

OBO ID: PO:0000055

IRI: http://purl.obolibrary.org/obo/PO_0000055

Definition: An undeveloped shoot system (PO:0009006).

reproductive bud

OBO ID: PO:0025084

IRI: http://purl.obolibrary.org/obo/PO_0025084

Definition: A bud that develops into a reproductive shoot system.

flower bud

OBO ID: PO:0000056

IRI: http://purl.obolibrary.org/obo/PO_0000056

Definition: A bud that develops into a flower.

inflorescence bud

OBO ID: PO:0000057

IRI: http://purl.obolibrary.org/obo/PO_0000057

Definition: A bud that develops into an inflorescence.

vegetative bud

OBO ID: PO:0000058

IRI: http://purl.obolibrary.org/obo/PO_0000058

Definition: A bud (PO:0000055) that develops into a vegetative shoot system (PO:0025607).

dormant leaf bud

OBO ID: PPO:0001009

IRI: http://purl.obolibrary.org/obo/PPO_0001009

Definition: A PO:'vegetative bud' (PO:0000058) that is participating in a GO:'bud dormancy process' (GO:0097207).

non-dormant leaf bud

OBO ID: PPO:0001010

IRI: http://purl.obolibrary.org/obo/PPO_0001010

Definition: A PO:'vegetative bud' (PO:0000058) that is not participating in a GO:'bud dormancy process' (GO:0097207).

breaking leaf bud

OBO ID: PPO:0001012

IRI: http://purl.obolibrary.org/obo/PPO_0001012

Definition: A 'non-dormant leaf bud' (PPO:0001010) that is in the 'bud burst stage' (PO:0025532).

Comment: If a breaking leaf bud is present, it implies that a new shoot system and unfolding true leaf are also present and observable, hence the “subclass of” axiom.

swelling leaf bud

OBO ID: PPO:0001011

IRI: http://purl.obolibrary.org/obo/PPO_0001011

Definition: A 'non-dormant leaf bud' (PPO:0001010) that is in the 'bud swell stage' (PO:0025531).

new shoot system

OBO ID: PPO:0001003

IRI: http://purl.obolibrary.org/obo/PPO_0001003

Definition: A 'vegetative shoot system' (PO:0025607) that does not yet have any unfolded true leaves (PPO:0001015).

new above-ground shoot-borne shoot system

OBO ID: PPO:0001004

IRI: http://purl.obolibrary.org/obo/PPO_0001004

new shoot system emerging from ground

OBO ID: PPO:0001005

IRI: http://purl.obolibrary.org/obo/PPO_0001005

new shoot system emerging from ground in first growth cycle

OBO ID: PPO:0001006

IRI: http://purl.obolibrary.org/obo/PPO_0001006

new shoot system emerging from ground in later growth cycle

OBO ID: PPO:0001008

IRI: http://purl.obolibrary.org/obo/PPO_0001008

vascular leaf

OBO ID: PO:0009025

IRI: http://purl.obolibrary.org/obo/PO_0009025

Definition: A leaf (PO:0025034) in a vascular plant.

Comment: Has vascular tissue. From APweb Glossary: In angiosperms, commonly thought of as one of the three basic parts of the seed plant body, a structure usually of determinate growth, without secondary thickening, and of superficial origin, often flattened and photosynthetic in part, and in the axil of which is found a bud. Occurs in the sporophytic phase of a plant life cycle.

cotyledon

OBO ID: PO:0020030

IRI: http://purl.obolibrary.org/obo/PO_0020030

Definition: A vascular leaf (PO:0009025) formed at the first shoot node (PO:0005004) of a plant embryo (PO:0009009) or a seedling (PO:0000003).

true leaf

OBO ID: PPO:0001013

IRI: http://purl.obolibrary.org/obo/PPO_0001013

Definition: A 'vascular leaf' (PO:0009025) that is not a 'cotyledon' (PO:0020030).

expanding true leaf

OBO ID: PPO:0001022

IRI: http://purl.obolibrary.org/obo/PPO_0001022

Definition: A 'true leaf' (PPO:0001013) that is in the 'vascular leaf expansion stage' (PO:0001052).

expanding unfolded true leaf

OBO ID: PPO:0001020

IRI: http://purl.obolibrary.org/obo/PPO_0001020

Definition: A 'expanding true leaf' (PPO:0001022) that is in the 'vascular leaf expanding unfolded stage' (PPO:0007006).

unfolding true leaf

OBO ID: PPO:0001014

IRI: http://purl.obolibrary.org/obo/PPO_0001014

Definition: A 'expanding true leaf' (PPO:0001022) that is in the 'vascular leaf unfolding stage' (PPO:0007005).

unfolded true leaf

OBO ID: PPO:0001015

IRI: http://purl.obolibrary.org/obo/PPO_0001015

Definition: A 'true leaf' (PPO:0001013) that is in the 'vascular leaf expanding unfolded stage' (PPO:0007006) or 'vascular leaf post-expansion stage' (PO:0001053) or 'vascular leaf senescent stage' (PO:0001054).

non-senescing unfolded true leaf

OBO ID: PPO:0001016

IRI: http://purl.obolibrary.org/obo/PPO_0001016

Definition: A 'unfolded true leaf' (PPO:0001015) that is in the 'vascular leaf expanding unfolded stage' (PPO:0007006) or 'vascular leaf post-expansion stage' (PO:0001053).

immature unfolded true leaf

OBO ID: PPO:0001018

IRI: http://purl.obolibrary.org/obo/PPO_0001018

Definition: A 'non-senescing unfolded true leaf' (PPO:0001016) that is in the 'vascular leaf expanding unfolded stage' (PPO:0007006) or 'vascular leaf expanded immature stage' (PPO:0007007).

expanded immature true leaf

OBO ID: PPO:0001021

IRI: http://purl.obolibrary.org/obo/PPO_0001021

Definition: A 'immature unfolded true leaf' (PPO:0001018) that is in the 'vascular leaf expanded immature stage' (PPO:0007007).

expanding unfolded true leaf

OBO ID: PPO:0001020

IRI: http://purl.obolibrary.org/obo/PPO_0001020

Definition: A 'expanding true leaf' (PPO:0001022) that is in the 'vascular leaf expanding unfolded stage' (PPO:0007006).

mature true leaf

OBO ID: PPO:0001019

IRI: http://purl.obolibrary.org/obo/PPO_0001019

Definition: A 'non-senescing unfolded true leaf' (PPO:0001016) that is in the 'vascular leaf mature stage' (PPO:0007008).

senescing true leaf

OBO ID: PPO:0001017

IRI: http://purl.obolibrary.org/obo/PPO_0001017

Definition: A 'unfolded true leaf' (PPO:0001015) that is in the 'vascular leaf senescent stage' (PO:0001054).

seed

OBO ID: PO:0009010

IRI: http://purl.obolibrary.org/obo/PO_0009010

Definition: A multi-tissue plant structure (PO:0025496) that develops from a plant ovule (PO:0020003) and has as parts a plant embryo (PO:0009009) enclosed in a seed coat (PO:0009088).

Comment: A seed generally develops from an ovule (PO:0020003) after fertilization, but may develop without fertilization in the case of apogamy (e.g., adventitious embryos or somatic embryos). A seed is a reproductive unit of seed plants (gymnosperms, angiosperms, and fossil pteridosperms).

mature seed

OBO ID: PPO:0001024

IRI: http://purl.obolibrary.org/obo/PPO_0001024

Definition: A 'seed' (PO:0009010) that has participated in a 'seed maturation stage' (PO:0007632).

whole plant

OBO ID: PO:0000003

IRI: http://purl.obolibrary.org/obo/PO_0000003

Definition: A plant structure (PO:0005679) which is a whole organism.

Comment: Examples include plant embryo (PO:0009009), megagametophyte (PO:0025279) and microgametophyte (PO:0025280).

herbaceous plant

OBO ID: PPO:0001000

IRI: http://purl.obolibrary.org/obo/PPO_0001000

perennial plant

OBO ID: PPO:0001002

IRI: http://purl.obolibrary.org/obo/PPO_0001002

plant embryo

OBO ID: PO:0009009

IRI: http://purl.obolibrary.org/obo/PO_0009009

Definition: A whole plant (PO:0000003) that participates in the plant embryo stage (PO:0007631).

Comment: A plant embryo is generally formed after the first division of a plant zygote (PO:0000423), but in the case of a nucellar (adventitious) plant embryo (PO:0004537), somatic plant embryo (PO:0025302), microspore-derived cultured plant embryo (PO:0025305), and other embryos that arise through apogamy, it begins after the division of a single cell that is not a zygote. The end of the embryo stage varies among taxa; the beginning of a seed germination stage (PO:0007057) in seed plants, formation of the first vascular leaf (PO:0009025) in pteridophytes, the beginning of development of a sporangium (PO:0025094) in bryophytes, or the beginning of the formation of a plant organ (PO:0009008) such as a root (PO:0009005), shoot axis (PO:0025029), or vascular leaf (PO:0009025) in a cultured plant embryo (PO:0000010).

seedling

OBO ID: PPO:0001007

IRI: http://purl.obolibrary.org/obo/PPO_0001007

woody plant

OBO ID: PPO:0001001

IRI: http://purl.obolibrary.org/obo/PPO_0001001

Data properties

The PPO defines four data properties that are used to record quantitive information about trait observations, such as the number of open flowers on a plant or the percent of leaves that are senescing. These data properties offer a great deal of flexibility in that they allow for numeric ranges, including open-ended ranges (e.g., "at least 10 fruits").

lower count

OBO ID: PPO:0000001

IRI: http://purl.obolibrary.org/obo/PPO_0000001

Definition: A relationship between a 'measurement datum' (IAO:0000109) and the inclusive lower endpoint of a non-negative integer range that contains the datum's value.

upper count

OBO ID: PPO:0000002

IRI: http://purl.obolibrary.org/obo/PPO_0000002

Definition: A relationship between a 'measurement datum' (IAO:0000109) and the inclusive upper endpoint of a non-negative integer range that contains the datum's value.

lower percent

OBO ID: PPO:0000003

IRI: http://purl.obolibrary.org/obo/PPO_0000003

Definition: A relationship between a 'measurement datum' (IAO:0000109) and the inclusive lower endpoint of a real number range within [0.0,100.0] that contains the datum's value.

upper percent

OBO ID: PPO:0000004

IRI: http://purl.obolibrary.org/obo/PPO_0000004

Definition: A relationship between a 'measurement datum' (IAO:0000109) and the inclusive upper endpoint of a real number range within [0.0,100.0] that contains the datum's value.
