# Supplementary material for: The Plant Phenology Ontology: A New Informatics Resource for Large-Scale Integration of Plant Phenology Data
Source: Front Plant Sci. 2018 May 1;9:517. doi: 10.3389/fpls.2018.00517 (PMC5938398; doi:10.3389/fpls.2018.00517)
Supplement: Supplementary file 2 [file Image_1.pdf]

Screenshots of unreasoned and reasoned representations of the PPO

Screenshots of the Protégé software showing partial class hierarchies for two representations of the PPO (Figures S.1 and S.2). First, for the PPO with only explicitly stated axioms (i.e., the “unreasoned” ontology, which is the file “ppo-raw.owl” in the release), and second, for the PPO after adding axioms inferred by the HermiT reasoner (i.e., the “reasoned” ontology, which is the file “ppo.owl” in the release). These screenshots can be reproduced by opening the released ontology files in Protégé version 5.0.0.

This document is supplementary material for: Stucky et al. (2018) The Plant Phenology Ontology: A new informatics resource for large-scale integration of plant phenology data. *Frontiers in Plant Science*. doi: 10.3389/fpls.2018.00517.

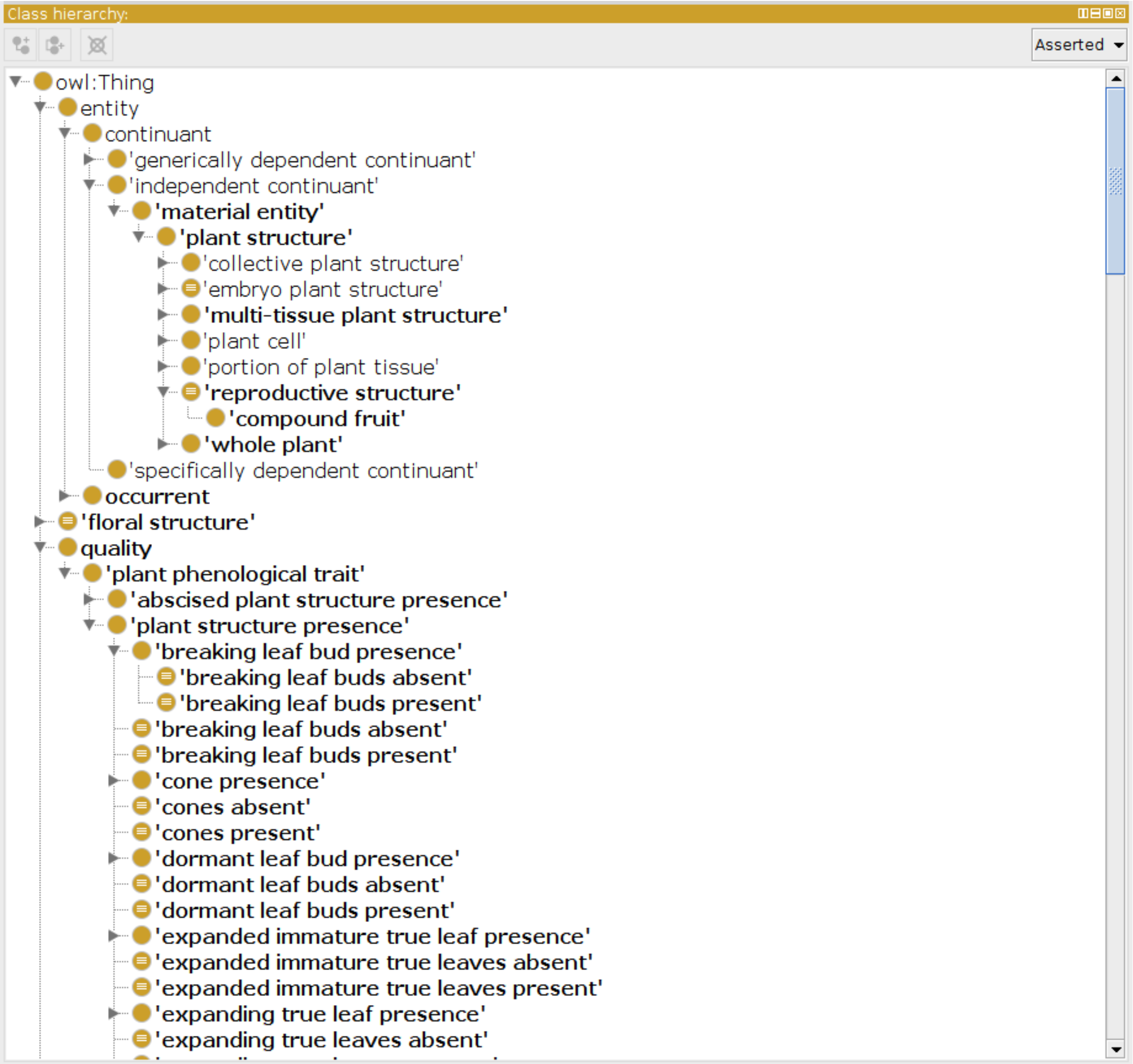

Figure S.1. Partial class hierarchy of the PPO prior to adding inferred axioms.

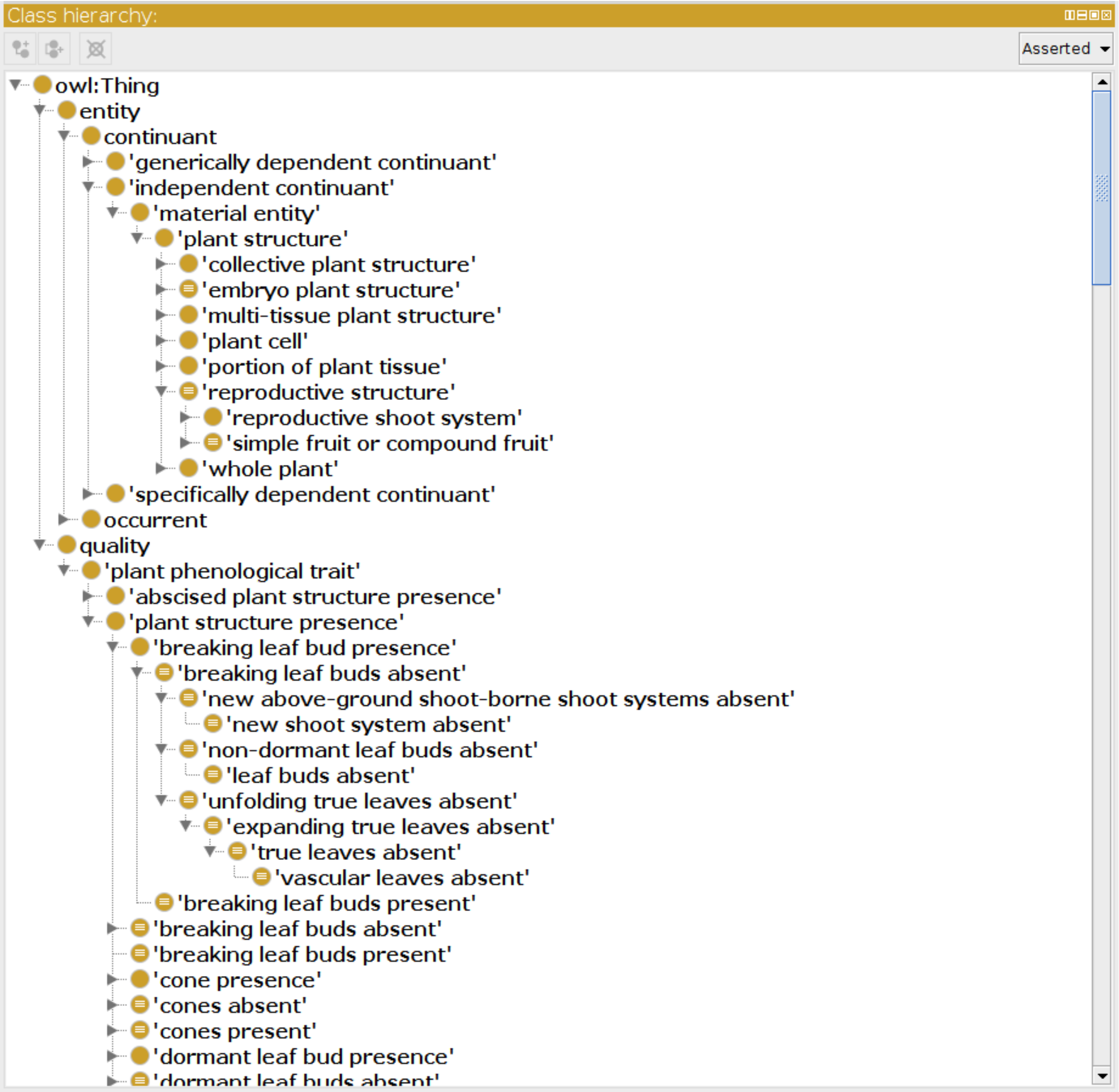

Figure S.2. Partial class hierarchy of the PPO after adding axioms inferred by the HermiT reasoner.
